# Non-contact vital-sign monitoring of patients undergoing haemodialysis treatment

**DOI:** 10.1038/s41598-020-75152-z

**Published:** 2020-10-28

**Authors:** Mauricio Villarroel, João Jorge, David Meredith, Sheera Sutherland, Chris Pugh, Lionel Tarassenko

**Affiliations:** 1 Department of Engineering Science, Institute of Biomedical Engineering, University of Oxford, Oxford, UK; 2 Oxford Kidney Unit, Oxford University Hospitals National Health Service Trust, Oxford, UK; 3 Nuffield Department of Medicine, University of Oxford, Oxford, UK

**Keywords:** Medical imaging, Applied optics, Optical techniques, Data acquisition, Data processing, Image processing

## Abstract

A clinical study was designed to record a wide range of physiological values from patients undergoing haemodialysis treatment in the Renal Unit of the Churchill Hospital in Oxford. Video was recorded for a total of 84 dialysis sessions from 40 patients during the course of 1 year, comprising an overall video recording time of approximately 304.1 h. Reference values were provided by two devices in regular clinical use. The mean absolute error between the heart rate estimates from the camera and the average from two reference pulse oximeters (positioned at the finger and earlobe) was 2.8 beats/min for over 65% of the time the patient was stable. The mean absolute error between the respiratory rate estimates from the camera and the reference values (computed from the Electrocardiogram and a thoracic expansion sensor—chest belt) was 2.1 breaths/min for over 69% of the time for which the reference signals were valid. To increase the robustness of the algorithms, novel methods were devised for cancelling out aliased frequency components caused by the artificial light sources in the hospital, using auto-regressive modelling and pole cancellation. Maps of the spatial distribution of heart rate and respiratory rate information were developed from the coefficients of the auto-regressive models. Most of the periods for which the camera could not produce a reliable heart rate estimate lasted under 3 min, thus opening the possibility to monitor heart rate continuously in a clinical environment.

## Introduction

It has been previously established that the colour and volume changes in superficial blood vessels during the cardiac cycle can be measured using a digital video camera. The use of a video camera can provide several advantages over traditional methods. It is non-invasive, it requires no skin preparation, causes no skin irritation and therefore can decrease the risk of infection. Moreover, no subject participation is required to set the equipment up. A camera-based solution has the potential to monitor patients at home.

Most of the current algorithms are based on methods for processing the photoplethysmography (PPG) waveform as recorded by a pulse oximeter, that is they attempt to record the blood volume changes associated with the cardiac cycle using a photo sensor. Hence, the term photoplethysmographic imaging (PPGi) is used throughout this paper. The basis for the estimation algorithms in most of the current methods involve computing the mean of the light intensity of pixels for each video frame and colour channel (red, green and blue) over one or more regions of interest (ROI) defined on the human face^1^. PPGi signals computed from different parts of the body such as the neck, arm or palm have also been analysed^2^. With the cost of digital video cameras continuing to decrease as the technology becomes more ubiquitous, research in non-contact vital-sign monitoring has greatly expanded in recent years. A summary of non-contact vital-sign monitoring methods can be found in^3,4^.

The standard vital signs monitored for patients in a hospital usually include temperature, heart rate (HR), respiratory rate (RR), blood pressure and, when appropriate, peripheral oxygen saturation ($SpO_2$). The routine measurement and interpretation of these vital signs can provide critical information about the underlying physiological state of patients. Most of the current work in video-based non-contact vital-sign monitoring has so far been performed over short time periods (typically up to a couple of minutes per recording) and under tightly controlled conditions with relatively still and healthy subjects. Using only a standard colour video camera and ambient light as the main illumination source, we propose data fusion algorithms to compute estimates of heart rate and respiratory rate from patients diagnosed with End-Stage Renal Disease (ESRD) undergoing haemodialysis treatment.

ESRD is the last stage of chronic kidney disease, occurring when the kidneys can no longer meet the daily demands to remove waste products and water from the body. Once severe kidney failure has been diagnosed, the patient will normally go through a form of dialysis treatment to remove the excess fluid and waste products from the blood. Haemodialysis is a common type of dialysis treatment. It uses a pair of surgically placed catheters (often located in the forearm) to circulate blood, at a low flow rate, from the body through an external dialysis machine known as dialyser. Waste products and toxins are removed by the dialysis machine and the clean blood is returned to the body. Although it can be carried out at home, haemodialysis is most often performed in a clinical centre. Patients attend the clinic two or three times a week for a treatment session lasting for approximately 4 h each visit. Patients usually lie on a bed or sit on a chair while connected to the dialysis machine^5^.

According to the last report from the United Kingdom Renal Registry published in 2016^6^, there were 61,287 patients who received dialysis or had a kidney transplant during 2015 in the UK, an 80% increase from 2000. Although kidney transplant was the most common treatment (53%), haemodialysis was still prevalent, accounting for 41% of the cases. Only 5% (1175) of the patients in haemodialysis received the treatment at home. ESRD is predominantly an adult disease^5^; the median age of patients was 67 years, 16% were aged 75+ years. Cardiovascular disease remained the primary cause of death in haemodialysis, accounting for 23% of the cases. Other leading causes of death were infection and treatment withdrawal, each accounting for approximately 21% of deaths^6,7^.

One of the advantages of recording videos from patients undergoing haemodialysis treatment is that, during the typical 4-h dialysis session, these patients experience a wide range of physiological values. This variability helps to validate vital-sign estimation algorithms.

## Results

A total of 84 dialysis sessions from 40 patients were recorded over the course of 1 year, as shown in Table 1. The total length of the video recorded was 304.1 h, with the average session lasting for approximately 3.5 h. The average age of the patients was 65 years. The majority of the patients were males (82.5%) with a mean body mass index (BMI) of 26.5. The patients were under dialysis treatment for an average of 30 months before being recruited to the study. As of March 2019, out of the 40 patients enrolled in the study, 27 had passed away (67.5%), 4 patients received a kidney transplant (10%), 1 patient was transferred to another dialysis unit, 7 continued to receive haemodialysis treatment in the hospital (17.5%) and only 1 patient recovered. The ethnicity of the patients was predominantly White British (87.5%). Only 3 patients were of Caribbean origin (7.5%), 1 patient from Asian background and 1 patient of a mixed background.

**Table 1:** Summary of the population in the clinical study.

Description	Value
Total number of patients	40
Total number of dialysis sessions	84
Total video length (h)	304.1
Video length per dialysis session (h)$^2$	3.5 (±0.9)
Age (years)$^2$	64.7 (±15.3)
Gender$^1$:	
Males	33 (82.5%)
Females	7 (17.5%)
Height (cm)$^2$	171.4 (±8.9)
Dry weight (kg)$^2$	77.1 (±15.3)
Body Mass Index (BMI)$^2$	26.5 (±5.4)
Ethnicity$^1$:	
White British	35 (87.5%)
Asian	1 (2.5%)
Caribbean	3 (7.5%)
Other mixed background	1 (2.5%)
Length on dialysis treatment (months)$^{2,3}$	30.2 (±23.3)

$^1$ N (percentage from total number of patients). $^2$ mean (± std). $^3$ Before the start of the study.

**Figure 1:**
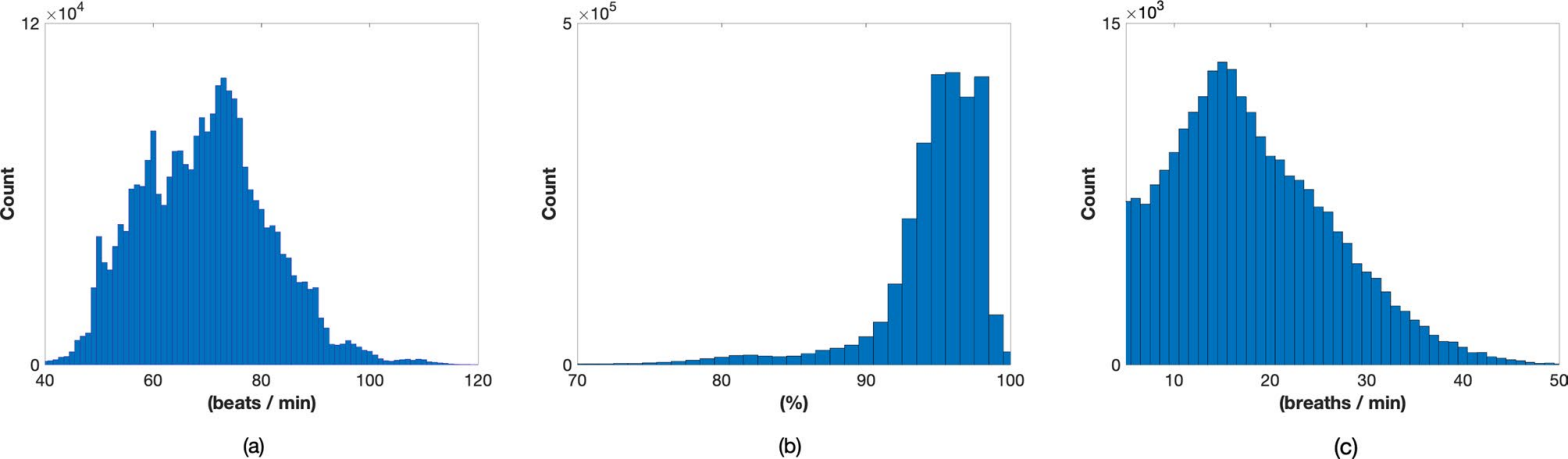
Distribution of physiological values for all the patients in the clinical study. (**a**) Heart rate and (**b**) $SpO_2$ from the finger pulse oximeter. (**c**) Respiratory rate from the chest belt.

Patients undergoing haemodialysis treatment present a wide range of physiological values. Figure 1 shows the distribution of heart rate, respiratory rate and $SpO_2$ measured with conventional monitoring equipment for all the patients in the study.

### Heart rate estimation

The first stage of our proposed methodology involved video processing algorithms to find suitable time periods during which the patient was stable, the location of the face was known and could be tracked. These algorithms were run on all 84 dialysis sessions. 23 sessions were judged to contain motion artefacts, unsuitable for heart rate estimation. This included periods of medical interventions or family visits during which the camera was moved away from the patient, video sessions with long time periods for which there was high patient activity, motion artefacts arising from the interaction between the patient and the clinical staff, or occurring when the patients were awake and engaging in conversation. Therefore, the resulting number of dialysis sessions (reported as *Valid camera data* column in Table 2) was 61, comprising a total video length of 219.8 h, representing approximately 72.3% of the total recording time. All 40 dialysis patients were represented in the resulting dataset.

Table 2 presents the overall heart rate estimation results for all the methods and data fusion techniques analysed. The *Estimated time* column reports the amount of good-quality data, with respect to the valid camera data, that was considered by each of the proposed algorithms to compute the heart rate estimates. The resulting *estimated time* varied depending on several factors, such as the beat-by-beat quality assessment of the PPGi signal extracted from the video or the differences between each of the signal processing algorithms and data fusion techniques proposed. The proportion of estimated time is reported relative to the valid camera data. The mean absolute error (MAE) and the mean absolute deviation (MAD) are reported over the total estimated time.

**Table 2:** Summary of the vital-sign estimation results for all recording sessions.

Vital sign	Data fusion	Data set/algorithm	Total recording time (h)	Valid camera data$^1$ (h, %)	Estimated time$^2$ (h, %)	$\hbox {MAE}^3$	$\hbox {MAD}^3$
Heart rate	Median	Beat count	304.1	219.8 h, 72.3%	93.0 h, 42.3%	2.1	2.2
		FFT	”	”	93.0 h, 42.3%	2.3	2.1
		AR dom pole	”	”	84.6 h, 38.5%	4.1	3.2
		ARk	”	”	113.6 h, 51.7%	2.6	1.9
	Kalman 1D	Beat count	”	”	134.3 h, 61.1%	2.3	2.9
		FFT	”	”	134.3 h, 61.1%	2.2	3.1
		AR dom pole	”	”	124.0 h, 56.4%	3.8	3.1
		ARk	”	”	139.4 h, 63.4%	2.9	2.1
	Kalman ND	Beat count	”	”	139.0 h, 63.2%	2.8	3.1
		FFT	”	”	139.0 h, 63.2%	3.4	4.1
		AR dom pole	”	”	131.0 h, 59.6%	4.1	4.2
		ARk	”	”	143.5 h, 65.3%	2.8	2.6
Respiratory rate		Face ROI	304.1	101.4 h, 33.3%	60.1 h, 60.3%	2.2	2.1
		Upper torso ROI	”	”	66.0 h, 65.1%	1.8	1.6
		All ROI combined	”	”	70.2 h, 69.2%	2.1	1.8

$^1$ Percentage with respect to the total recording time. $^2$ Percentage with respect to the valid camera data. $^3$ beats/min for HR and breaths/min for RR.

**Figure 2:**
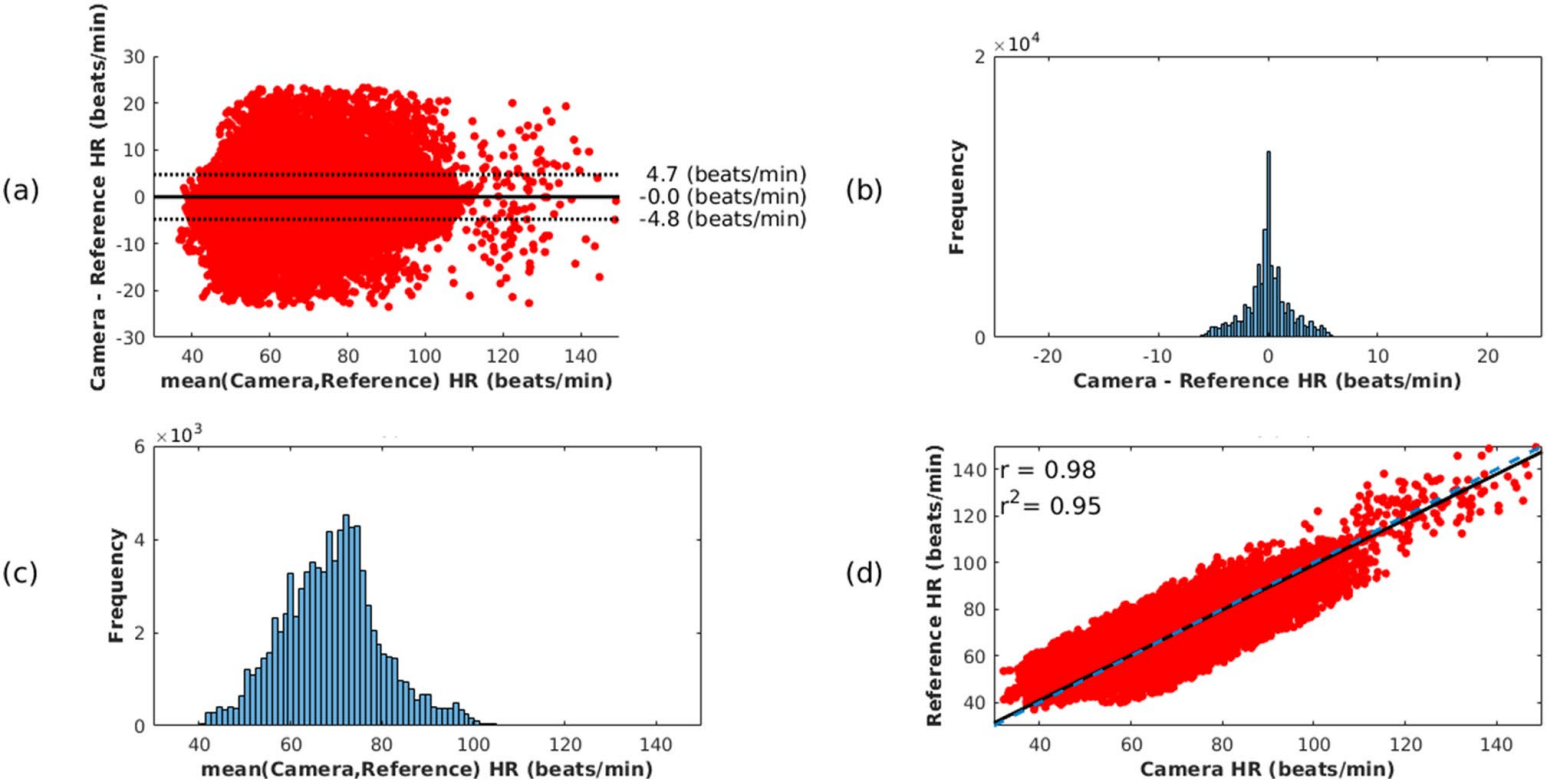
Agreement between the reference heart rate values (computed from the agreement between the two pulse oximeters) and the camera estimates (computed using the ARk algorithm and the Kalman ND data fusion method) for the valid dialysis video data, comprising a total recording time of approximately 219.8 h. (**a**) The Bland–Altman plot presents no sensor bias. (**b**) The differences between the camera estimates and the reference heart rate values are normally distributed. (**c**) The distribution of the mean values shows that most of the heart rate estimates are within the expected physiological range for adults. (**d**) The plot shows a strong correlation between the two devices, with a correlation coefficient of 0.98.

Figure 2 shows in more detail the agreement between the reference heart rate values and the video camera estimates (using the ARk algorithm and the Kalman ND data fusion method) for all the valid dialysis video data, comprising a total of recording time of 219.8 h. The Bland–Altman plot, shown in Fig. 2a, presents no sensor bias with narrow differences across the heart rate physiological range, implying a good agreement between the two measurements. The differences, shown in Fig. 2b, are normally distributed. The distribution of the mean values in Fig. 2c, shows that the values are within the expected physiological range for adults. The values estimated by the two devices have a positive correlation coefficient of 0.98, as shown in Fig. 2d.

### Respiratory rate estimation

**Figure 3:**
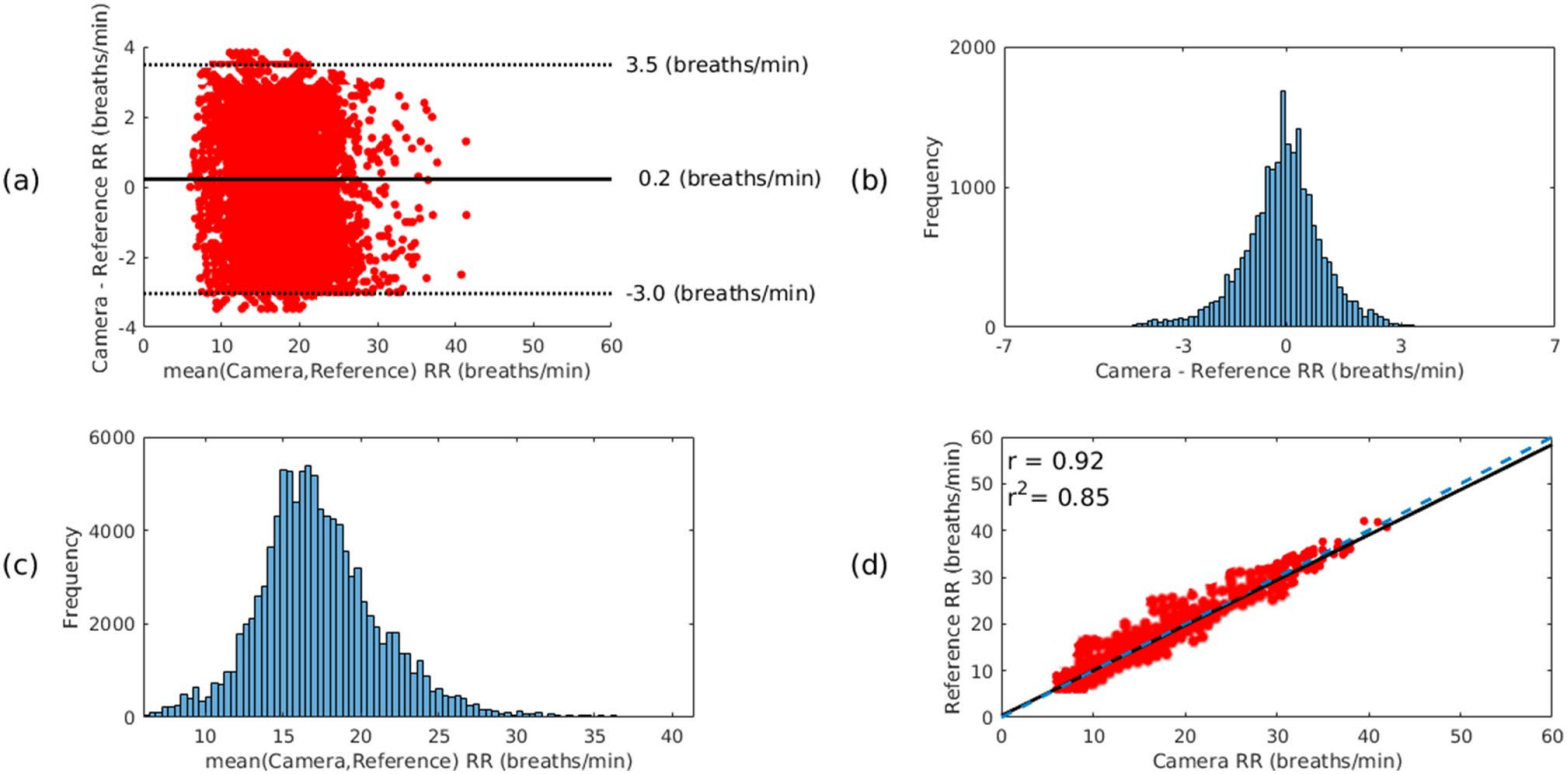
Agreement between the reference respiratory rate values (computed from the ECG and chest belt) and the camera estimates computed over the regions of interest on the patients’ face for the valid dialysis video data, comprising a total recording time of approximately 101.4 h. (**a**) The Bland–Altman plot presents minimal sensor bias. (**b**) The differences between the two estimates. (**c**) The distribution of the mean values show that most of the respiratory rate estimates are within the expected physiological range for adults. (**d**) The plot shows a strong correlation between the two devices, with a correlation coefficient of 0.92.

According to the analysis of the reference data set (see supplementary methods 2), the total time the reference respiratory rate, as computed from the Electrocardiogram (ECG) and chest belt, agree within 2 breaths per minute is 101.4 h. Figure 3 shows in more detail the agreement between the reference respiratory rate values and the video camera estimates computed over the regions of interest on the patients’ face for the valid dialysis video data. The Bland–Altman plot, shown in Fig. 3a, presents minimal sensor bias with narrow differences across the respiratory rate physiological range, implying a good agreement between the two measurements. The distribution of differences between the two measurements, shown in Fig. 3b, is also normally distributed. The distribution of the mean values in Fig. 3c, shows that the values are within the expected physiological range for adults. The values estimated by the two devices have a positive correlation coefficient of 0.92, as shown in Fig. 3d.

Table 2 compares the MAE and MAD values for the three respiratory rate estimation strategies over the valid dialysis video data with the corresponding proportion of data estimated.

## Discussion

The recording of patient data for a clinical study within a hospital presents many challenges, especially the recording of the reference physiological signals (heart rate and respiratory rate in the case of this paper) against which the camera estimates are compared and evaluated. Most of the studies reported in the literature use only one data source or reference device as the gold standard. For example, the use of one pulse oximeter to record a single set of heart rate values, without an extensive analysis of the quality and accuracy of the reference device signals. With this in mind, our clinical study was designed to have at least two reference data sources for heart rate and respiratory rate.

The haemodialysis population in our study was mostly comprised of the elderly, with an average age of 64.7 years (see Table 1). Most of the patients were diagnosed with illnesses such as diabetes, hypertension and other autoimmune diseases. Patients were also given medications such as calcium-channel blockers to regulate blood pressure by relaxing and widening blood vessels. Other medications included Amlodipine, an angiotensin II receptor antagonist used to help regulate blood pressure and heart rate to slow the progression of kidney disease. These, and other medications, potentially affected the patients’ physiology, and as a consequence, the values recorded by the pulse oximeters.

The heart rate values, as provided by the pulse oximeters, occasionally presented large errors of more than 50 beats/min as shown in supplementary figure 1 in supplementary methods 1. The pulse oximeter attached to the ear often underestimated the heart rate values with respect to the pulse oximeter attached to the finger. From the 304.1 h for which simultaneous pulse oximetry recordings were available, only 79.7% (approximately 242.7 h) of the values differed by no more than 5 beats/min (see supplementary table 1 in supplementary methods 1), as recommended by accepted clinical standards^8^.

The appropriate monitoring of heart rate is vital to the physiological assessment of the patient’s health status. In an Intensive Care Unit (ICU) environment, heart rate is measured continuously typically on a second-by-second basis by using the ECG as the gold standard method^9^. On other hospital wards, heart rate is measured less frequently. For non-critical patients, it is often measured manually by the nurses or other clinical staff. Evans et al.^10^ analysed a large set of publications raising concerns that there is limited guidance on the frequency at which vital signs should be recorded, concluding that much of the information is based on surveys of nurses, clinical practice reports and expert opinions. For example, Schumacher^11^ found that the frequency of the recording of vital signs in post-operative patients varied between 15, 30 and 60 min, and occasionally once every 4 h. Botti and Hunt^12^ interviewed nurses and found that vital signs were measured every 30 min for the first 4 h following discharge from the recovery unit. Moreover, 86% of nurses have been reported to underestimate heart rate measurements in patients with atrial fibrillation^13^.

**Figure 4:**
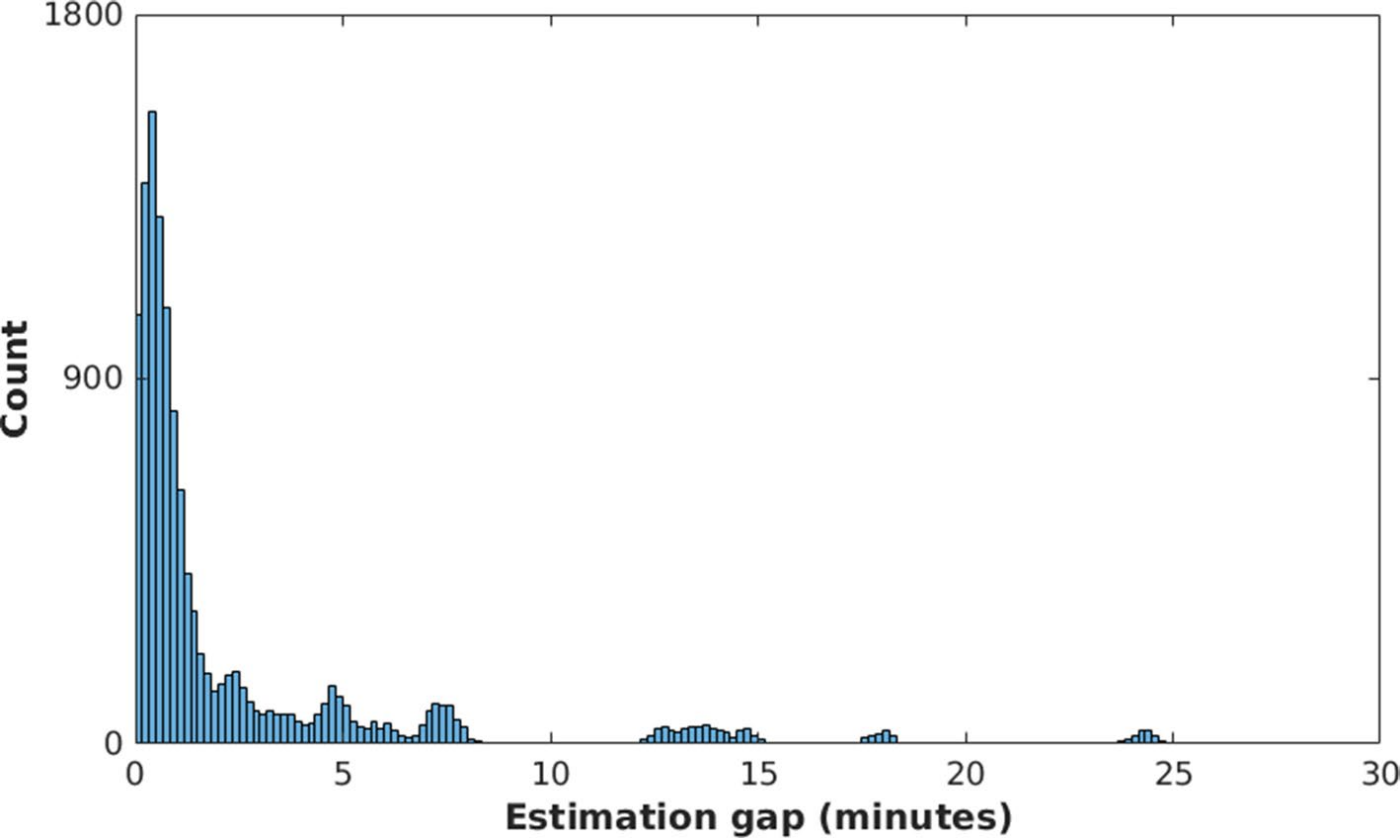
Gaps in heart rate estimation using the Kalman ND data fusion method. Most of the periods for which the camera could not produce a heart rate estimate were under 3 min.

Recording video from ill patients in a healthcare centre, such as a hospital or a clinic, presents unique challenges that makes the continuous beat-by-beat estimation of vitals signs difficult. In our study, there were time periods during which the pulsatile signal extracted from the video camera was corrupted and, therefore, heart rate estimates could not be reliably computed. Figure 4 shows the distribution of the time gaps between heart rate estimates from the video camera for the entire dialysis data set. The majority of gaps between the estimates were under 3 min, with the most common gap lasting for 30 s. Although some longer gaps occurred occasionally in the dialysis population, our results are comparable to our previous clinical studies involving the monitoring of vital signs of preterm infants in the Neonatal Intensive Care Unit (NICU)^14^. We reported that the majority of time periods for which heart rate could not be computed in the NICU were less than 30 s.

The reasons for the camera not providing a reliable heart rate estimate were largely due to motion artefacts and the interactions between the clinical staff and the patients. As opposed to the ICU, where patients are often sedated and resting, the patients undergoing haemodialysis treatment enrolled in our study were usually awake and engaging in conversation with nurses, visiting family members or were free to use media devices such as mobile phones. Although heart rate estimates were not provided for every second of the entire recording session, most of the periods for which the camera could not produce a reliable heart rate value were under 3 min. This opens the possibility to monitor heart rate with a clinically relevant frequency, with respect to the current clinical practice of manual observations for non-critical patients^10–12^.

Most of the current work in video-based non-contact vital-sign monitoring has so far been performed over short time periods of typically up to a couple of minutes per recording session, under tightly-controlled and well-lit conditions with relatively still and healthy subjects^3,4^. This has several implications on the choices of equipment to use and how the videos are recorded. The video cameras used in other studies are usually placed very close to the subjects. The physiology of the healthy volunteers enrolled doesn’t have large variability, with reference heart rates typically around 60 *beats*/*min*. As such, these studies typically use off-the-shelf video cameras, record video using a common video container (such as .avi or .mpeg) and a common compression codec (such as H.264 or motion JPEG).

One of the goals of our study was to obtain pulsatile signals from the video camera data with sufficient quality that would allow us to compute reliable vital signs estimates in a real-world hospital setting. There were several factors that were considered when designing the recording setup used in our clinical study. The camera was positioned at least 1 metre away from the subjects. This was required so that the clinical staff in the hospital could have rapid access to the patients in case of an emergency or to perform the regular clinical care plan. During the typical 4-h haemodialysis treatment session, patients presented a wide variation of their recorded vital signs. As shown in Fig. 6, the amplitude of the pulsatile waveforms extracted from the video camera data, from which heart rate estimates were computed, was very small. The quality of the recorded video was negatively affected by the typical daytime changes in the lighting conditions of the hospital ward, from bright well-lit summer days to dark winter days. Changes in the intensity of artificial light sources (such as fluorescent lighting), the shadows created by the constant movement of the clinical staff across the hospital ward, and light reflected from electronics devices reduced the ability of the camera to capture the small colour changes related to the cardiac cycle from the subject’s skin. Therefore, we developed custom software to record video from a high-resolution camera using an uncompressed, RAW video format.

Unlike other studies reported in the literature, the video camera and the recording format chosen for our clinical study required a substantial amount of storage. However, this was only required for the research phase to develop the algorithms. It is expected that a system implemented in the hospital, would not need to store any of the videos, it would only need to store the vital sign estimates. This can can help the integration of camera-based solutions with current hospital information systems.

Further research and analysis is needed to establish guidelines for the optimal type of video camera to use in a clinical setting. Some factors to consider are the quality of the lighting environment available in the hospital ward, the field of view required, the distance at which the camera would be set and the number of patients to be simultaneously monitored from a single camera. If only one patient is required to be monitored at a close distance in a well-lit scenario, such as a camera on top of a neonatal incubator to monitor infants in the NICU^14^, a low-resolution off-the-shelf camera can potentially be used. However, the further the camera is placed from the patient(s), the higher the requirements of the quality of the camera will be. The established clinical workflow and any policies required to protect the privacy of the patients, clinical staff or any other individuals are factors that might affect the recording of video and the option of video camera system to use.

The proposed algorithms in this paper were developed for the retrospective analysis of the video data. A real-time analysis was beyond the scope of this work. However, our prototype algorithms, implemented in the MatLab programming language, were able to run at a rate of 240 frames per second (FPS). As a proof of concept, we have also implemented a real-time prototype of some of our algorithms to estimate heart rate and respiratory rate using the video cameras available in regular mobile phones. This application is available to download from^15^.

Video cameras are an attractive choice for non-contact monitoring of vital signs due to their ubiquity, high performance and low cost. Non-contact vital-sign monitoring provides several advantages over traditional methods because no subject participation is required to set the equipment up. It requires no skin preparation, causes no skin irritation, decreasing the risk of infection. It has the potential to be seamlessly integrated not only into the patient’s care in the hospital or clinical practice, but also into the patient’s lifestyle at home. Non-contact vital sign monitoring technology can augment and complement existing technologies in use in the hospitals, such as patient monitors recording the ECG. Camera-based monitoring technologies could not only estimate vital signs, but also could provide other physiological assessments such as pain levels, motor development and neurological status from the analysis of facial expressions and patient activity.

## Methods

### Clinical study

The Oxford University Hospitals Trust is one of the largest teaching trusts of the National Health Service (NHS) in the United Kingdom. The research adhered to the NHS Code of confidentiality and Oxford University standards. Ethical approval was granted by the Oxford University Clinical Trials and Research Governance (Reference # 11/SC/0207).

#### Study design

The study was designed to run in parallel with regular dialysis treatment sessions, which minimised the inconvenience to patients of having to give additional time for research purposes. No patient care decisions were made on the basis of the data collected during the study. The interventions in the study were minimally invasive and similar in nature to those occasionally undertaken when more intensive monitoring is used with patients who are acutely unwell. As our study required a degree of exposure to place electrodes and monitoring probes for the reference measurements, patient dignity and privacy were respected at all times. A precise description of which interventions were planned, and what these would entail, was discussed with the patient before the start of any involvement in the study.

Ethical approval was granted to study 40 patients. The research was compliant with the relevant government and regulations of the Oxford University NHS Foundation Trust. The collection of personal data met the requirements of the Data Protection Act. Individual informed written patient consent was obtained from all the participants in the study to record the videos and publish the results, including anonymised images. Each patient was recorded multiple times to offset any potential data loss from patients moving out of the area to other Renal Units or from patients receiving a kidney transplant during the study period and, therefore, not able to complete the recordings. Even though patients consented to participate in the study and agreed to be recorded for the entire dialysis session, periods for which a patient did not wish to be recorded were encountered. Common time periods of privacy concern included medical interventions, family visits or other personal reasons of the patient. Therefore, the clinical staff were allowed to move the camera away or stop video recording if the patient requested.

The following individuals were excluded from the study: Patients under 18 years old, patients unable to tolerate continuous monitoring, patients scheduled for kidney transplant and patients participating in other studies that might lead to confounding effects on either study.

**Figure 5:**
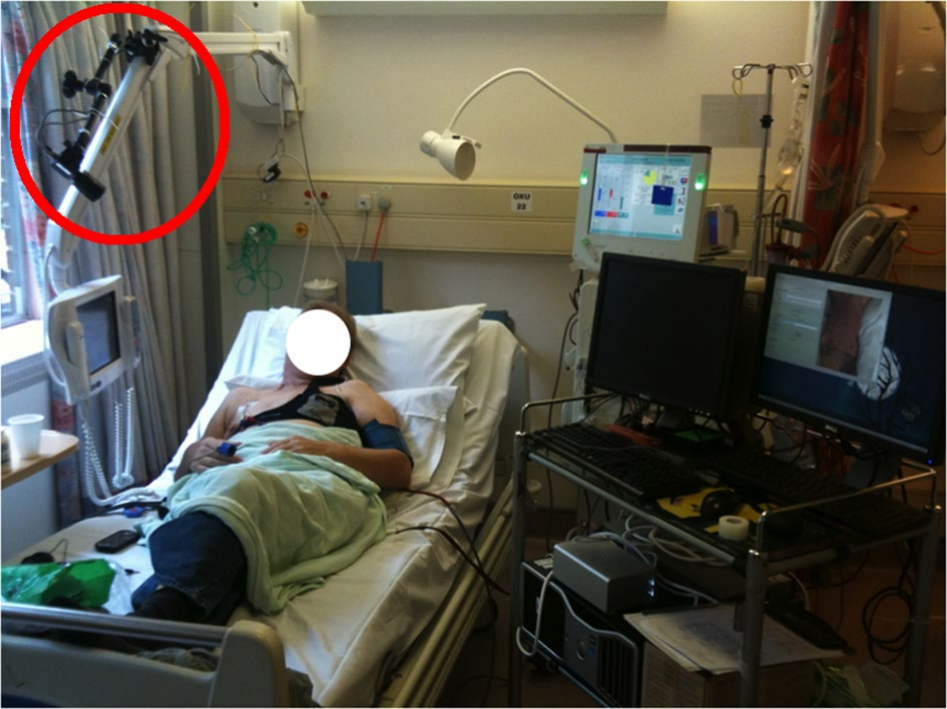
A typical dialysis data collection set-up. The red circle shows the location of the video camera. A pulse oximeter is attached to the patient’s finger on the right hand. The Equivital EQ-02 LifeMonitor is located on the patient’s chest. Consent was obtained from the patient to use the image.

#### Instrumentation

Figure 5 shows the typical data collection set-up used for the study. Two reference devices in regular clinical use were chosen as the primary means to correlate the results of the analysis with the data extracted from the video recordings: Hidalgo’s Equivital EQ-02 LifeMonitor (Equivital, Hidalgo, Cambridge, UK), shown as a belt harness attached to the patient’s chest; and a separate Bluetooth pulse oximeter (Model 4100, Nonin Medical, Plymouth, MN, USA), typically attached to the patient’s finger on the right hand.

The Equivital EQ-02 LifeMonitor is a certified ambulatory vital-sign device intended for the monitoring of adults. It consists of a chest belt harness which contains skin electrodes and a thoracic expansion sensor. It records two-channel Electrocardiogram (ECG) waveforms at a sampling frequency of 256 Hz and a respiration signal (from the chest belt) at 25 Hz. The sensor also provides two respiratory rate estimates, computed from the chest belt and ECG respectively, at a rate of one estimate every 5 s. The manufacturer customised the device to add a wired interface for an external pulse oximeter module (Nonin OEM-III) that was attached to the patient’s ear lobe. The pulse oximeter module provided estimates of heart rate and $SpO_2$ at a rate of 1 estimate every 5 s.

The second reference device, the Nonin pulse oximeter model 4100, is a clinically-approved device. In collaboration with the manufacturer, the authors developed customised software for the acquisition of a PPG signal at a sampling rate of 75 Hz, in conjunction with 4-beat averages of heart rate and $SpO_2$ at a sampling rate of 3 Hz.

#### Video recording

The Grasshopper2 camera (Point Grey Research, Richmond, Canada) was used in the study. The camera can record 3 colour channels: red, green and blue (RGB) at a maximum rate of 15 frames per second (FPS). The video camera was positioned at approximately 1 m away from the patient (see red circle in Fig. 5). A Fujinon HF12.5SA-1 lens (Fujifilm Holdings Corporation, Tokyo, Japan) was attached to the camera to ensure most of the upper torso of the patient was visible. Video was recorded in uncompressed RAW video format at 15 frames per second, 24-bit colour (8-bit for each colour channel), at a resolution of 2448$\times$2048 pixels. A typical 4-h dialysis session required close to 3 terabytes of storage. This presented some challenges for the recording, data integrity and long-term archival of the database. Therefore, the authors developed custom data acquisition software following international standards; so that the system, with minor modifications, may be submitted for medical software certification in the future.

### Overview of the proposed framework

The proposed vital-sign estimation system begins by analysing the input video to identify suitable time periods for which the location of the patient within the video frame can be detected and tracked, discarding periods for which high activity or motion artefacts occur. Heart rate and respiratory rate are subsequently estimated using data fusion algorithms by analysing several regions of interest (ROI) across the patient’s body such as the face or chest.

### Video processing

One of the requirements for our clinical study was not to interfere with regular patient care. As opposed to an intensive care unit (ICU), where patients are often sedated and resting, patients under haemodialysis treatment are usually awake and active. As a result, the video recordings were affected by several sources of distortion, including: regular interaction between the patients and clinical staff, motion artefacts due to patient discomfort, equipment malfunction, the use of media devices such as mobile phones or other external factors outside of our control. In several cases, the video camera was moved away from the patient to ease access for a medical intervention or to preserve the privacy of visiting family members who wished not to appear in the video frame.

#### Face detection and tracking

Video analysis started with the task of detecting and tracking the patient’s face. Several algorithms have been proposed in the literature using cues such as skin colour, face or head shape, facial appearance or a combination of more than one of them. Although these problems have received a lot of attention, they are still challenging, especially when illumination, subject expression and object occlusion vary considerably. Furthermore, the process of detecting and tracking the location of the patient from videos recorded in a hospital presents other unique challenges due to the nature of the environment and the regular clinical activities being carried out.

Three face detectors were developed based on^16,17^: one for frontal faces, one for approximately 3/4 view to full left-profile and one for right-profile faces. The face detectors were trained on a dataset consisting of 300 hand-labelled images from each of the 40 patients divided into three groups: frontal, left-profile and non-face images. Each group contained a total of 4000 sample images. Right-profile faces were trained using the left-profile images flipped horizontally. The face detectors were run in parallel for all video frames. Feature points were computed for all the faces detected in each of the images. For any pair of faces found in consecutive frames, the number of common feature points normalised by the number of feature points which were not shared by both faces, was used as an overlap score. If the overlap score was greater than 0.5, a match was declared and the two faces were connected.

#### Selection of stable time periods

Suitable time periods for estimating heart rate and respiratory rate from the video camera data were defined as video during which there was minimal patient movement. Our clinical study was designed so that the distance between the patient and the camera was similar for most of the recorded videos. Changes of the centroid ($C_x$, $C_y$) of the detected face over time was used as an indicator of the degree of subject motion. Motion *M*(*i*) at frame *i* was defined as the Euclidean distance, measured in pixels, between centroids for two successive frames:

1 $$\begin{aligned} \text {M}(i) = \sqrt{\big (C_{x}(i) - C_{x}(i-1)\big )^{2} + \big (C_{y}(i) - C_{y}(i-1)\big )^{2}}. \end{aligned}$$

Subject motion was quantified as a binary activity index on a frame by frame basis, defined by:

2 $$\begin{aligned} Act(i) = {\left\{ \begin{array}{ll} 0, &{} \ \text {if }\ \text {M}(i)>100 \text { pixels}\\ 1, &{} \ \text {otherwise} \end{array}\right. } \end{aligned}$$

**Figure 6:**
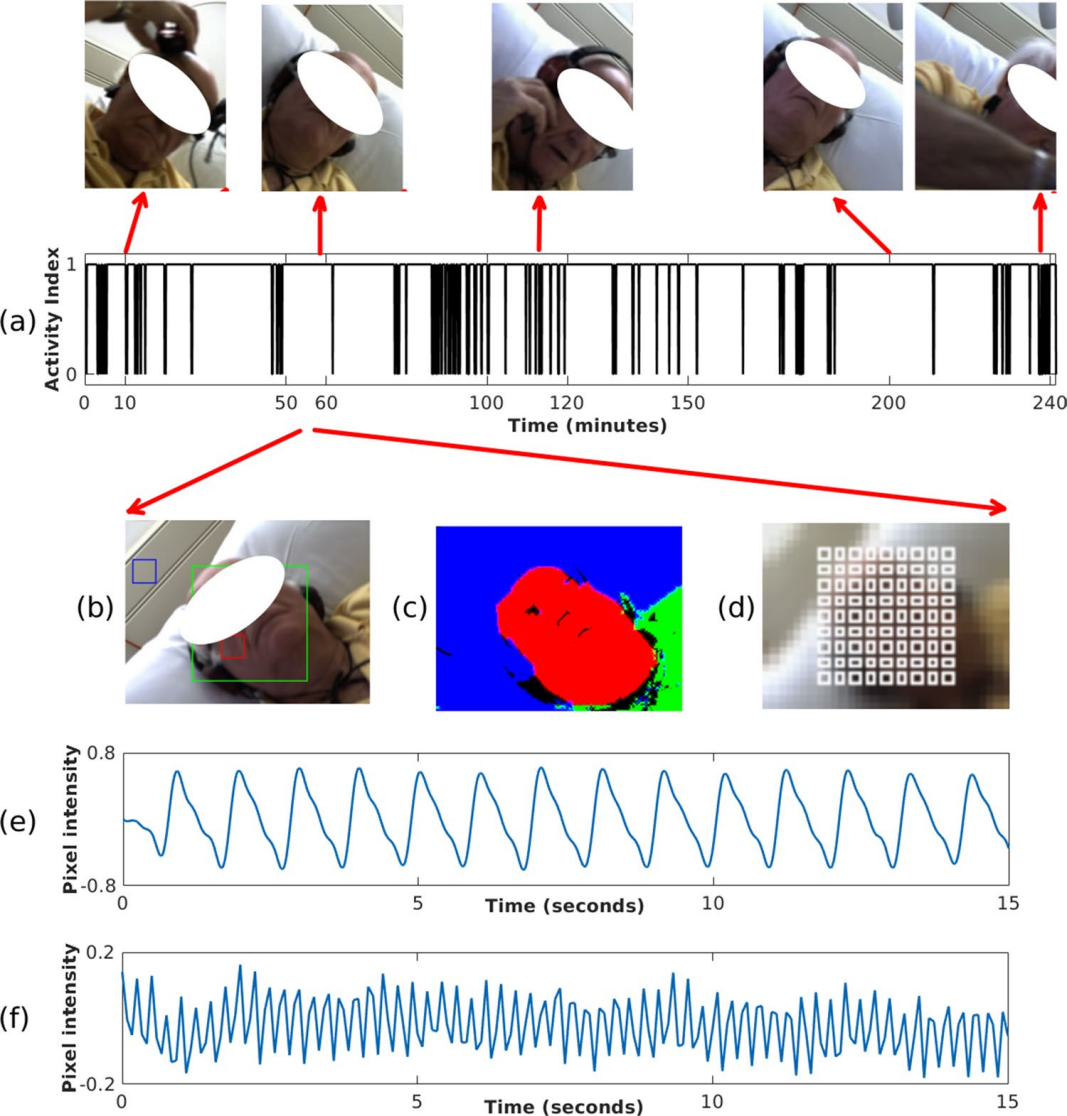
The process of extracting PPGi signals. (**a**) Activity Index computed for a typical 4-h dialysis session with screenshots above showing the patient: adjusting headphones to listen to music near minute 10, sleeping at minute 60, awake and engaging in a phone conversation at minute 120, sleeping at minute 200, and interacting with the clinical staff at minute 240. (**b**) The image at minute 60 showing: the patient’s detected face (green rectangle), $ROI_R$ (blue square), and a sample $ROI_S$ (red square). (**c**) Image segmentation output. (**d**) Location and size of the grid for all $ROI_S$. (**e**) A sample 15-s PPGi time series extracted from the $ROI_S$ in (**b**). (**f**) Sample $ROI_R$ time series extracted from the $ROI_R$ in (**b**). Consent was obtained from the patient to use these images.

When the Euclidean distance between the centroids of the detected patient’s face in two successive frames was above a threshold of 100 pixels, the patient was considered as being active and the frame was assigned an activity index of 0, corresponding to a time period of high patient motion. If instead the distance was below the threshold, the frame was assigned a value of 1, corresponding to a stable time period suitable for vital-sign estimation. Figure 6a shows the result of computing the activity index for a typical 4-h dialysis session, with sample images corresponding to quiet periods during sleep and periods during which the patient was active.

Video sessions for which the proportion of suitable time periods was less than one-third of the total length of the video were discarded and no further analysis was performed. Vital signs were estimated using the remaining video sessions.

#### Image segmentation

Once the location of the patient in the video frame was identified, a non-parametric Bayesian image segmentation^18^ algorithm was used to segment the images into three areas: face, upper body and background (see Fig. 6b,c). These three areas were used by algorithms to select regions of interest from which heart rate and respiratory rate can be estimated.

A Dirichlet process mixture model (DPMM) for image histogram clustering was used for the image segmentation process. A Markov Random Field (MRF) method was applied after segmentation for automatic model selection under smoothness spatial constraints.

### Reference physiological values

The reference heart rate estimates in our study were provided by two transmission-mode pulse oximeters, one located on the finger and the other on the ear lobe. Similarly, two reference respiratory rate estimates were provided by the manufacturer, estimated from the ECG and chest belt. Important information about the peripheral circulation can be derived by simultaneously recording vital signs from different body sites. However, the use of multiple devices to record the physiology from hospital patients, as opposed to young healthy volunteers, might introduce several factors to be considered before their estimates can be used as gold-standard measurements.

When two sensing devices are used to measure a physiological phenomenon, neither provides an absolute correct measurement. A direct comparison (on a sample-by-sample basis) of the heart rate or respiratory rate values between the video camera and each of the reference values provided by the manufacturers, can potentially be affected not only by physiological factors but also by the recording set-up and internal processing context for each manufacturer. This could lead to incorrect performance results. In similar scenarios, the average of the measurements from two devices or methods is usually taken as the representative values^19^. Thus, we propose that new robust reference heart rate and respiratory rate can be obtained by analysing the source signals recorded by the manufacturers. These new gold-standard heart rate values were then used to compare the estimates computed from the video camera. A more detailed description about the process of computing the reference heart rate and respiratory rate is presented in supplementary methods 1 and 2 provided for this paper.

### Heart rate estimation

One PPGi signal was extracted for each of the camera’s three colour channels from several ROI’s defined over an area covering the patient’s face. We then used algorithms which we developed to assess the quality of the cardiac pulses found in each PPGi signal. Heart rate was subsequently estimated from each PPGi signal individually using four time-domain and frequency-domain algorithms. The overall heart rate estimate for the patient was finally computed by combining the individual estimates from each of the previous four methods using three data fusion techniques.

#### PPGi extraction

Two types of ROI were defined for every image frame: Subject and Background regions of interest. Subject regions of interest are image areas for which heart rate can be measured by analysing the varying colour of the reflected light. A total of 81 regions, labelled $\{ROI_{S,1}, \ldots , ROI_{S,81}\}$, were organised in a 9x9 grid and laid out evenly covering the area of the patient’s face (see Fig. 6d). One background region of interest, labelled $ROI_R$, was placed on an area not containing any body parts from the patient (typically the background wall) to analyse the common effects of external lighting sources on the skin and background (see Fig. 6b,c).

The average pixel intensity in each image frame was computed for every colour channel from every ROI. The resulting time series from all the $ROI_S$ are known as PPGi signals. The PPGi time series contain both high and low frequency components that make heart rate estimation challenging. To eliminate frequencies outside the physiological heart rate range, chosen to be from 36 to 240 beats/min, the signals were detrended and a finite-impulse response (FIR) band-pass filter was applied with cut-off frequencies of 0.6 Hz and 4 Hz. Figure 6e, f show 15-s signals extracted from the subject $ROI_S$ and background $ROI_R$ respectively.

#### Identifying step changes

**Figure 7:**
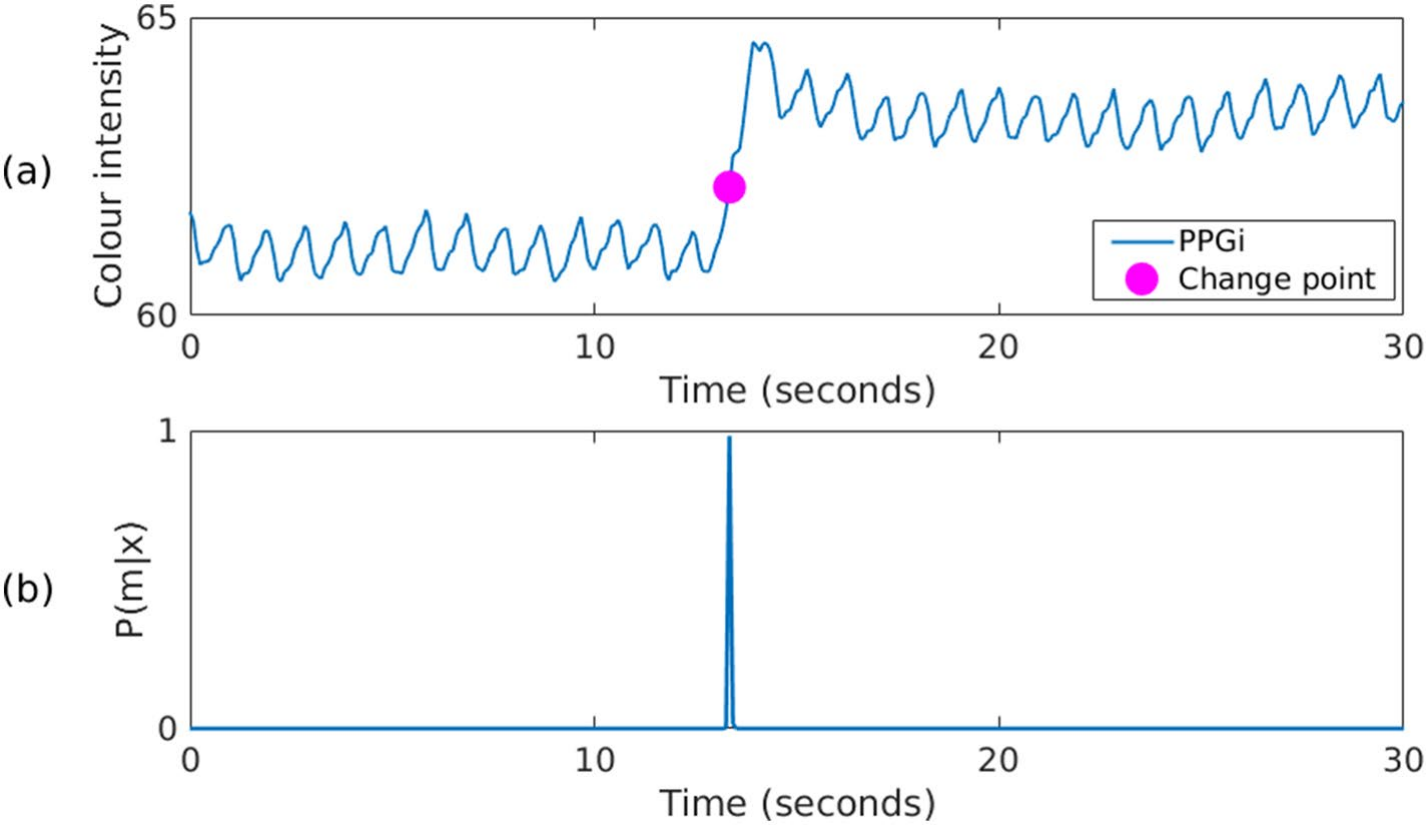
Bayesian change point detection algorithm applied to a 30-s window during which a fluorescent light was turned on, producing a step change in the recorded pixel intensity signal. (**a**) Input PPGi signal with the change point marked around second 13. (**b**) The probability of the change point.

Even during periods for which patients were quiet (sleeping or listening to music), abrupt variations or discontinuities in the PPGi signals occurred. These step changes, or change points, were often caused by sudden shifts in the overall lighting (ambient lights turned on or off) or by the effects of shadows cast by the clinical staff walking around the patient bed. A typical illumination step change is shown in Fig. 7, corresponding to a time when a fluorescent light was turned on.

To estimate the occurrence of these step changes, a Bayesian change point detection algorithm based on^20,21^ was applied to the PPGi signal. Given a data sequence *x* of *N* samples from two piece-wise constant inputs $\mu _1$ and $\mu _2$ with Gaussian noise added:

3 $$\begin{aligned} x_i = {\left\{ \begin{array}{ll} \mu _1 + \epsilon _i, &{} \text {if } i<m.\\ \mu _2 + \epsilon _i, &{} \text {otherwise}. \end{array}\right. } \end{aligned}$$

where the noise samples $\epsilon _i$ are assumed to be independent. The likelihood of the data is given by the joint probability of the noise samples $\epsilon _i$:

4 $$\begin{aligned} P(x|\{\mu _1\mu _2\sigma m\}) = \prod \limits _{i=1}^N P(\epsilon _i) \end{aligned}$$

where $\sigma$ is the standard deviation of the Gaussian noise; $\mu _1, \mu _2$ and $\sigma m$ are the known time series parameters. The probability density function for the noise samples is defined by:

5 $$\begin{aligned} P(\epsilon ) = \frac{1}{\sigma \sqrt{2 \pi } } e ^{ - \frac{ (\epsilon - \mu )^2 }{2\sigma ^2} } \end{aligned}$$

Substituting Eq. (5) into Eq. (4), the likelihood is given by:

6 $$\begin{aligned} P(x|\{\mu _1\mu _2\sigma m\}) = (2\pi \sigma ^2)^{-\frac{N}{2}} exp\left[ - \frac{1}{2\sigma ^2} \left( \sum _{i=1}^{m} (x_i - \mu _1)^2 + \sum _{i=m+1}^N (x_i - \mu _2)^2 \right) \right] \end{aligned}$$

Given Bayes’ theorem, the posterior probability density of the signal parameters ${\mu _1\mu _2\sigma m}$ given the data sequence *x* can be computed by:

7 $$\begin{aligned} P(\{\mu _1\mu _2\sigma m\}|x) = \frac{P(x|\{\mu _1\mu _2\sigma m\}) P(\{\mu _1\mu _2\sigma m\})}{P(x)} \end{aligned}$$

Assuming all parameter values are equally likely a priori and by integrating over the parameters $\mu _1, \mu _2$ and $\sigma m$ , the posterior probability density can be modified to account only for the location of the step change *m*^22^. Therefore, the probability of the step change at location *m* given the data sequence *x* is computed by:

8 $$\begin{aligned} P(m|x) \ \propto \quad \frac{1}{\sqrt{m(N-m)}} \left[ \sum _{i=1}^N x_i^2 - \frac{1}{m} (\sum _{i=1}^m x_i)^2 - \frac{1}{N-m}(\sum _{i=m+1}^N x_i)^2 \right] ^{-\frac{N-2}{2}} \end{aligned}$$

Several step changes can occur during video recording. The change point detection algorithm was applied to 30-s running windows with 5-s overlap.

#### Beat detection

**Figure 8:**
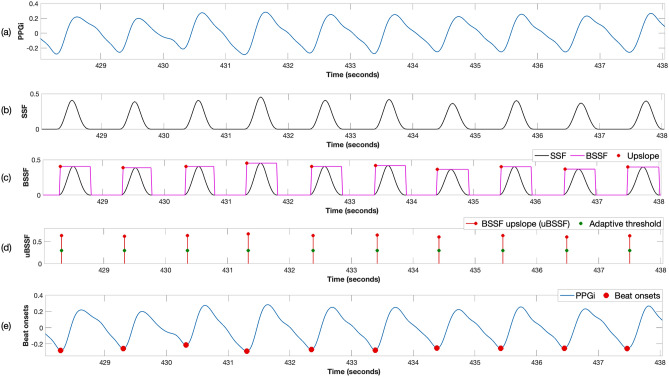
Steps to compute the location of the onset of each cardiac pulse for a 10-s PPGi time series: (**a**) Input PPGi waveform. (**b**) Slope sum function (SSF). (**c**) Boxed Slope Sum Function (BSSF), scaled to match each pulse in the SSF function. (**d**) The adaptive threshold applied to the uBSSF signal. (**e**) The detected beat onsets.

There are several beat detection algorithms proposed in the literature. The large amount of data to process from all the PPGi signals extracted for the entire duration of the video recordings required an optimised approach. An extension of the algorithms proposed by Zong et al.^23^ was developed. Given a PPGi time series $x_i = x_1, x_2, \ldots x_n$ (see Fig. 8a), a new time series labelled the slope sum function (SSF) was computed over a running window that contained only the positive upslopes of each pulse:

9 $$\begin{aligned} SSF_i = \sum _{k=i-w}^{i} u_k \qquad ;\quad u_k = {\left\{ \begin{array}{ll} \Delta x_k = x_k - x_{k-1}, &{}\ \Delta x_k > 0 \\ 0, &{}\ \Delta x_k \le 0 \\ \end{array}\right. } \end{aligned}$$

where *w* is the length of the window, approximately 170 ms, which corresponds to the duration of the upslope of the PPG signal for an adult with a normal resting heart rate of 60 beats/min. An example of the SSF time series can be seen in Fig. 8b.

Subsequently, a Boxed Slope Sum Function (BSSF) was computed by finding the zero-crossing points at the beginning of the upslope and at the end of the downslope for each pulse in the SSF signal (Fig. 8c). The upslope points from the BSSF signal were extracted and labelled as uBSSF. To find the beat onsets, instead of analysing the amplitudes of all the values in the SSF signal, as in^23^, only the uBSSF signal was processed. An initial amplitude threshold was computed as the median of the first 30 s from the uBSSF data points. This threshold value was adaptively updated as the uBSSF values increased or decreased. For each subsequent uBSSF data point, if its value was greater than the computed threshold, the data point was considered as a beat onset and the threshold was increased by 10% of the difference between the previous threshold and the current uBSSF data point. If the uBSSF data point was below the threshold, the threshold was reduced by 10% (Fig. 8d). The peak of each PPGi pulse was computed by finding the location of the maximum point between two detected beat onsets (Fig. 8e).

#### Iterative multi-scale dynamic time warping

The algorithms we proposed in this paper to estimate heart rate from the video camera data, required the PPGi signal to be regular for short-time periods of a typical duration between 15 and 30 s (see Fig. 8e). This is required so that spectral analysis could be performed on a given time window to compute the heart rate. However, heart rate in individuals is not constant, it varies during the day depending on the kind of work the body is doing at any given moment. This phenomenon is called heart rate variability. Heart rate variability is often used as a tool for assessing the integrity of the autonomic nervous system, the interaction between psychological states and autonomic control, and the pathophysiology of diseases that involve or are influenced by autonomic function^24^.

To assess how regular the cardiac pulses found in the video camera data are, each heart beat in the PPGi signal was compared with a running 30-s pulse template using an iterative multi-scale dynamic time warping (DTW) algorithm. DTW is well suited for this type of pattern-matching problem^25^, its goal is to compute an optimal alignment path with minimal overall cost (warping path) between two time series that do not necessarily have the same length. The PPGi signal was subdivided into 30-s running windows with an overlap of 5 s. For each window, a pulse template was constructed from all the beats within the window. If a template could not be computed, the window was considered invalid, the beats within the window were flagged as poor-quality and no further processing was done.

The window template was computed in three stages. In the initial stage, the template was constructed by including only the pulses with a beat-to-beat interval corresponding to a heart rate between 36 to 240 beats/min. During the second stage, a new template was computed by including only those beats for which the cross-correlation coefficient with the template of the first stage was greater than 0.8. Finally, in the third stage, a template was constructed by including all the beats with an amplitude less than three times the standard deviation computed from the valid beats in the previous stage. This amplitude criterion helped to prevent the window template from including large oscillations due to motion artefacts. For every stage, all the pulses were aligned using their peaks as reference points and a template was computed as the average of all valid beats according to the given criterion. The length of the template was taken as the median of all the beats within the window. The method proceeded to the next stage only if at least 50% of the pulses were deemed to be valid. If the method was successful in all stages, the template of the third stage was taken as the window template. Once a valid template was computed for a given window, the template was compared to each of the beats within the window using the proposed iterative multi-scale DTW algorithm.

**Figure 9:**
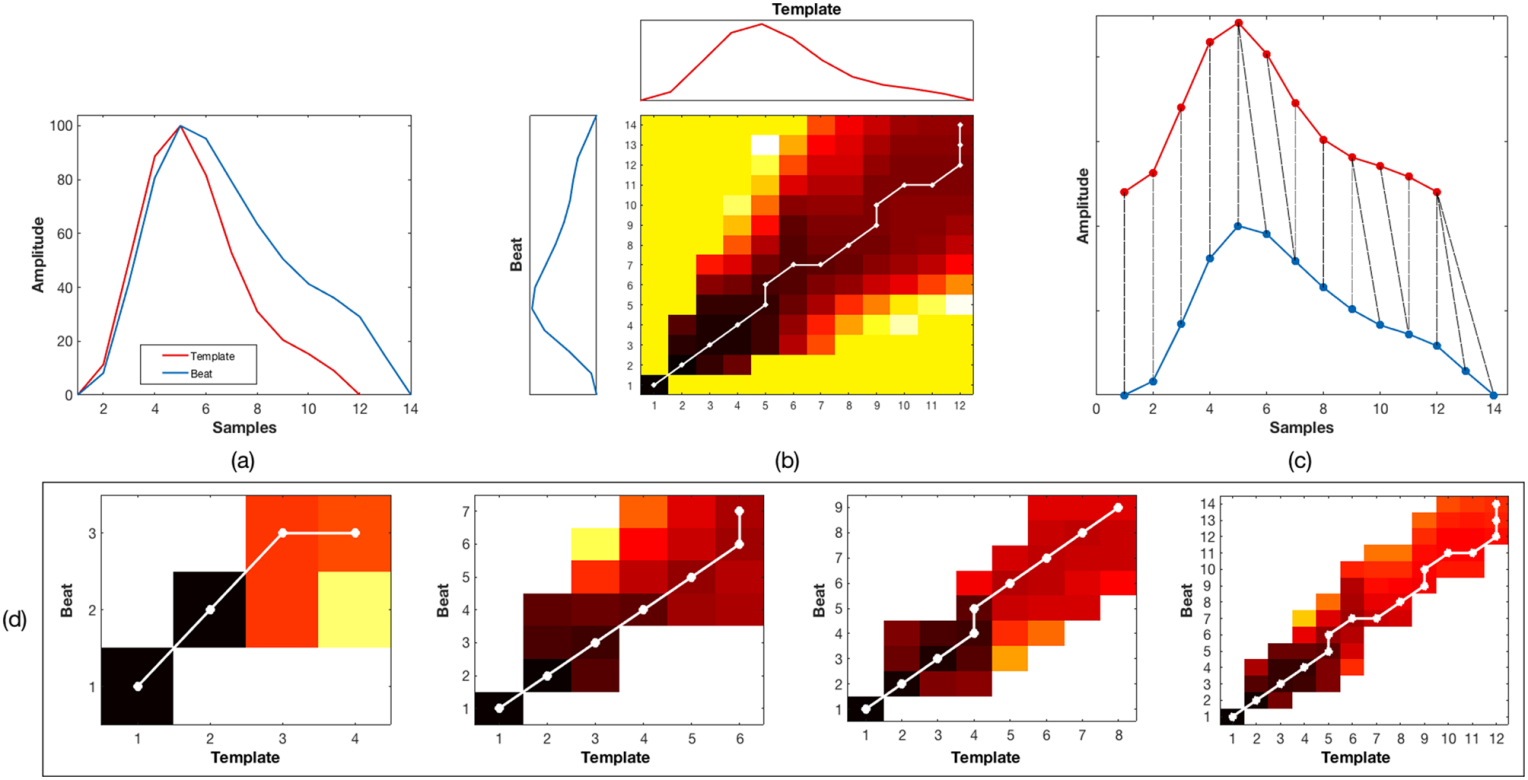
Time alignment for multi-scale DTW. (**a**) Normalised input beat (blue) and template (red). (**b**) The accumulated distance matrix with darker regions present areas where the beat and template are close, while brighter regions constitute areas where they are further apart; the optimal minimum path is shown in white. (**c**) DTW time alignment between the beat and template. (**d**) Finding the minimum path in the iterative multi-scale DTW algorithm.

Given that the quality of all the PPGi waveforms from all the ROI had to be assessed, an improvement on the classical DTW algorithm is proposed based on the work done by^26,27^. Two time series $X = x_1, x_2, \ldots , x_n$ of length *N* and $Y = y_1,y_2,\ldots ,y_m$ of length M were defined, representing the current beat to be tested and the window template respectively. To reduce the effects of short-term upward or downward trends in the PPGi signal, the current beat and template were rotated and their amplitude normalised (see Fig. 9a). Instead of the commonly used Manhattan distance, we defined a local cost function *c*(*x*, *y*) based on the difference between the beat and template slopes. This function was defined so that it has a small value (low cost) when *x* and *y* are similar to each other, and has a large value (higher cost) when *x* and *y* are different.

In classical DTW, a cost matrix $C \in \mathfrak {R}^{N \times M}$ is obtained by evaluating the local cost function for each pair of elements of the time series *X* and *Y*, as shown in Fig. 9b. The total cost $c_p(X,Y)$ of any warping path *p* of length *L* is then typically given by:

10 $$\begin{aligned} c_p(X,Y) = \sum _{l=1}^L c(x_{nl},y_{ml}) \end{aligned}$$

The optimal minimal cost path usually runs through a valley of low cost values (darker areas) within the cost matrix, as shown in Fig. 9b. The time alignment for the current PPGi beat and the window template is shown in Fig. 9c. To reduce the number of cells for which to compute the cost matrix *C*(*X*, *Y*), time alignment was done on downsampled versions of the time series *X* and *Y* at different scales based on a piece-wise linear approximation method. At each scale, the lengths *N* and *M* were decreased and the optimal path found. An optimal warping path between *X* and *Y* is a warping path $p^*$ with minimal total cost among all possible warping paths. Therefore, the DTW distance *DTW*(*X*, *Y*) at a given scale was defined as:

11 $$\begin{aligned} DTW(X,Y) = c_{p^*}(X,Y) = min \{c_p(X,Y)\} \end{aligned}$$

Once the optimal path with minimal cost was found at a given scale, it was then projected onto the next scale. The search for a new optimal path at the next scale was only carried at within boundaries defined by the previous scale (see Fig. 9d). The overall minimal distance between the beat *X* and the corresponding window template *Y* was found from the last DTW scale.

#### Beat-by-beat quality assessment

Once the beat onsets and peaks were detected, the quality of each pulse in the PPGi signal was evaluated based on the work by^28^ and expanded in^29^. The PPGi signal was subdivided into 30-s running windows with an overlap of 5 s. For the *k*-th beat ($b_k$) in each window, several Signal Quality Indices (SQI) were computed that measured: the degree of motion of the patient, the occurrence of step changes, inter-beat interval outside a physiological range, the changes in the amplitude of the PPGi signal, the clipping of the amplitude of the PPGi signal, and finally, a similarity metric based on the DTW algorithm presented in the previous section.

Beats corresponding to time periods during which there was a high degree of patient movement, as identified by the activity index in Eq. (2), were flagged as invalid by assigning a value of 0 to the activity quality index $SQI_{\mathrm{act}}$:

12 $$\begin{aligned} SQI_{\mathrm{act}}(b_k) = {\left\{ \begin{array}{ll} 0, &{} \ \text {if any frame}\ i\ \in b_k, \ \text {Act}(i) = 0 \\ 1, &{} \ \text {otherwise} \end{array}\right. } \end{aligned}$$

If the probability of a step change to occur in any of the image frames for given beat was greater than $80\%$, as defined by the change point detection algorithm in Eq. (8), the beat was flagged as invalid by assigning to the change point quality index $SQI_{\mathrm{cp}}$ a value of 0:

13 $$\begin{aligned} SQI_{\mathrm{cp}}(b_k) = {\left\{ \begin{array}{ll} 0, &{} \ \text {if any frame}\ i\ \in b_k, \ \text {P}(i|x) > 0.8 \\ 1, &{} \ \text {otherwise} \end{array}\right. } \end{aligned}$$

The next quality metric assessed whether the inter-beat time intervals were within a physiological range corresponding to a heart rate between 36 and 240 beats/min. This range was considered as safe limits for patients in our dataset (see Fig. 1) and for adult patients in hospital care or at home, since some clinical practices are starting to include exercise training programs as part of the treatment of haemodialysis patients^30,31^. The frequency bounding quality index $SQI_{\mathrm{freq}}$ was taken to be 0 if the instantaneous heart rate $HR_{\mathrm{inst}}$ of the *k*-th beat fell outside the valid physiological range:

14 $$\begin{aligned} SQI_{\mathrm{freq}}(b_k) = {\left\{ \begin{array}{ll} 0, &{} \ \text {if}\ \ HR_{\mathrm{inst}}(b_k) < 36\ \ \text {and}\ \ HR_{\mathrm{inst}}(b_k) > 240\\ 1, &{} \ \text {otherwise} \end{array}\right. } \end{aligned}$$

Sudden spikes in the amplitude of the PPGi signal occurred during the recording. These were often caused by small head movements, speech or changes in the light intensity recorded by the video camera caused by artificial light sources. The $SQI_{\mathrm{amp}}$ was set to 0 if the amplitude of the *k*-th beat was outside three standard deviations $\sigma _w$ from the mean $\mu _w$ of its corresponding window *w*:

15 $$\begin{aligned} SQI_{\mathrm{amp}}(b_k) = {\left\{ \begin{array}{ll} 0, &{} \ \text {if any frame}\ i\ \in b_k, \ \ PPGi(i) <\mu _w - 3\cdot \sigma _w \quad \text {or} \quad PPGi(i) > \mu _w + 3\cdot \sigma _w \\ 1, &{} \ \text {otherwise} \end{array}\right. } \end{aligned}$$

Clipping or saturation of the light intensity recorded by the video camera often occurred as a result of motion artefacts or during bright lighting conditions. Signal clipping can be detected when the derivative of the signal crosses a given threshold^32^. Given that $N_{\mathrm{length}}(b_k)$ is the length of the *k*-th beat and $N_{\mathrm{clipped}}(b_k)$ is the proportion of the derivative of the *k*-th beat that crosses a clipping threshold of 0.1, the $SQI_{\mathrm{clip}}$ was set to 0 when more than one-third of the derivative was clipped:

16 $$\begin{aligned} SQI_{\mathrm{clip}}(b_k) = {\left\{ \begin{array}{ll} 0, &{} \ \text {if}\ \ N_{\mathrm{clipped}}(b_k)/N_{\mathrm{length}}(b_k)\ >\ 1/3\\ 1, &{} \qquad \text {otherwise} \end{array}\right. } \end{aligned}$$

The last quality metric, $SQI_{\mathrm{dtw}}$, was computed to assess how regular the cardiac pulses found in the PPGi signal were. $SQI_{\mathrm{dtw}}$ is a continuous metric, varying between 0 and 1, comparing each *k*-th beat to the average pulse of its corresponding window using the DTW method presented in the previous section:

17 $$\begin{aligned} SQI_{\mathrm{dtw}}(b_k) = 1 - DTW(X, Y)/100 \end{aligned}$$

Once all the individual SQI metrics were computed, deciding if ultimately a beat was of good or poor quality involved a simple decision rule. The combined SQI ($SQI_{\mathrm{beat}}$) for each *k*-th beat was derived by:

18 $$\begin{aligned} SQI_{\mathrm{beat}}(b_k) = SQI_{\mathrm{act}}(b_k) \cdot SQI_{\mathrm{cp}}(b_k) \cdot SQI_{\mathrm{freq}}(b_k) \cdot SQI_{\mathrm{amp}}(b_k) \cdot SQI_{\mathrm{clip}}(b_k) \cdot SQI_{\mathrm{dtw}}(b_k) \end{aligned}$$

**Figure 10:**
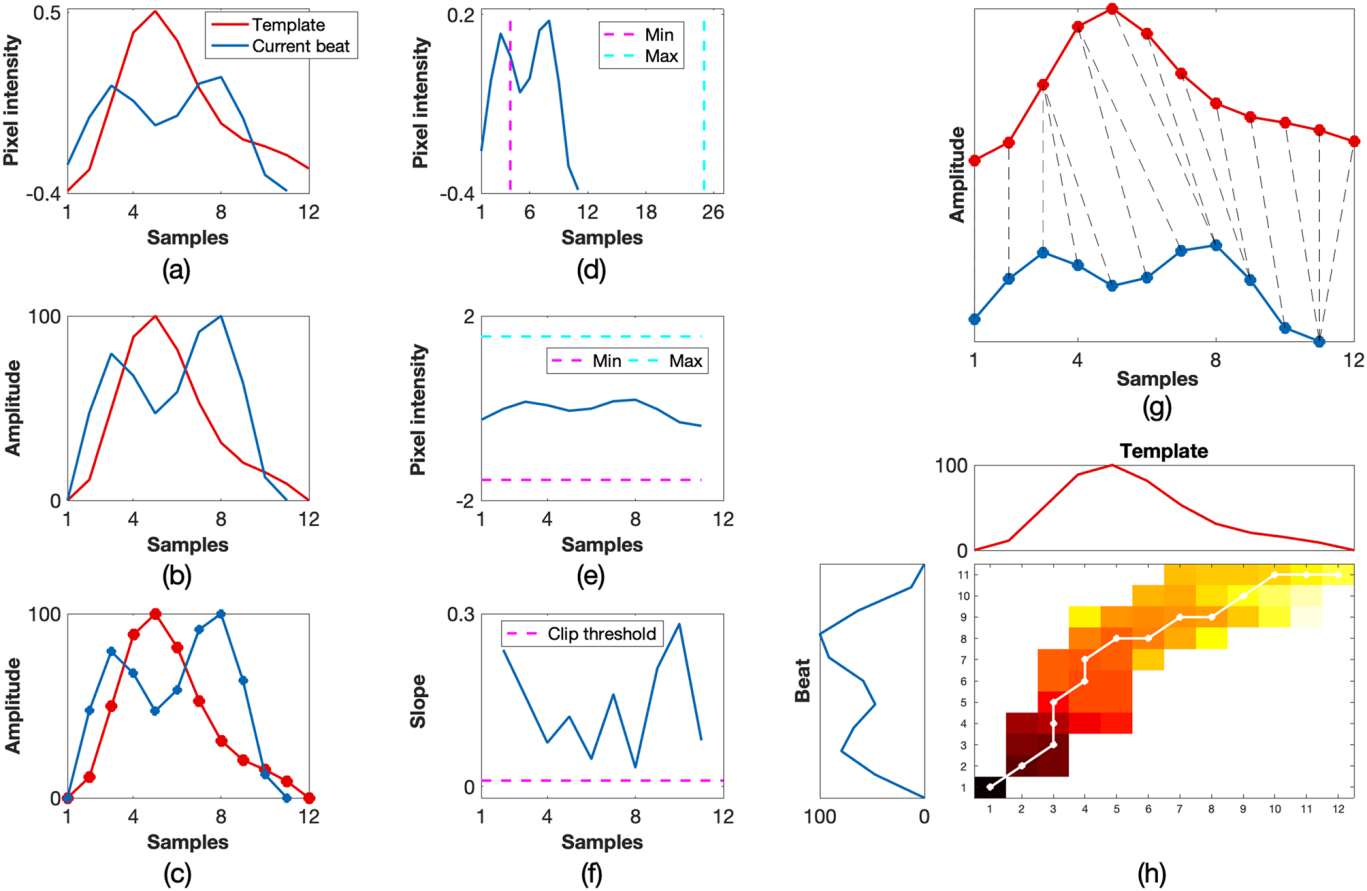
Example of a poor-quality beat. The resultant combined $SQI_{\mathrm{beat}}$ value was 0. (**a**) Input template and beat, (**b**) rotated and normalised template and beat, (**c**) piece-wise linear approximation, (**d**) check if the beat was within physiological bounds for heart rate, (**e**) check if the beat was within the amplitude thresholds for the current window, (**f**) clipping detection, (**g**) DTW showing the minimum path with a cost of 28.10, (**h**) accumulated cost matrix showing the minimum path.

**Figure 11:**
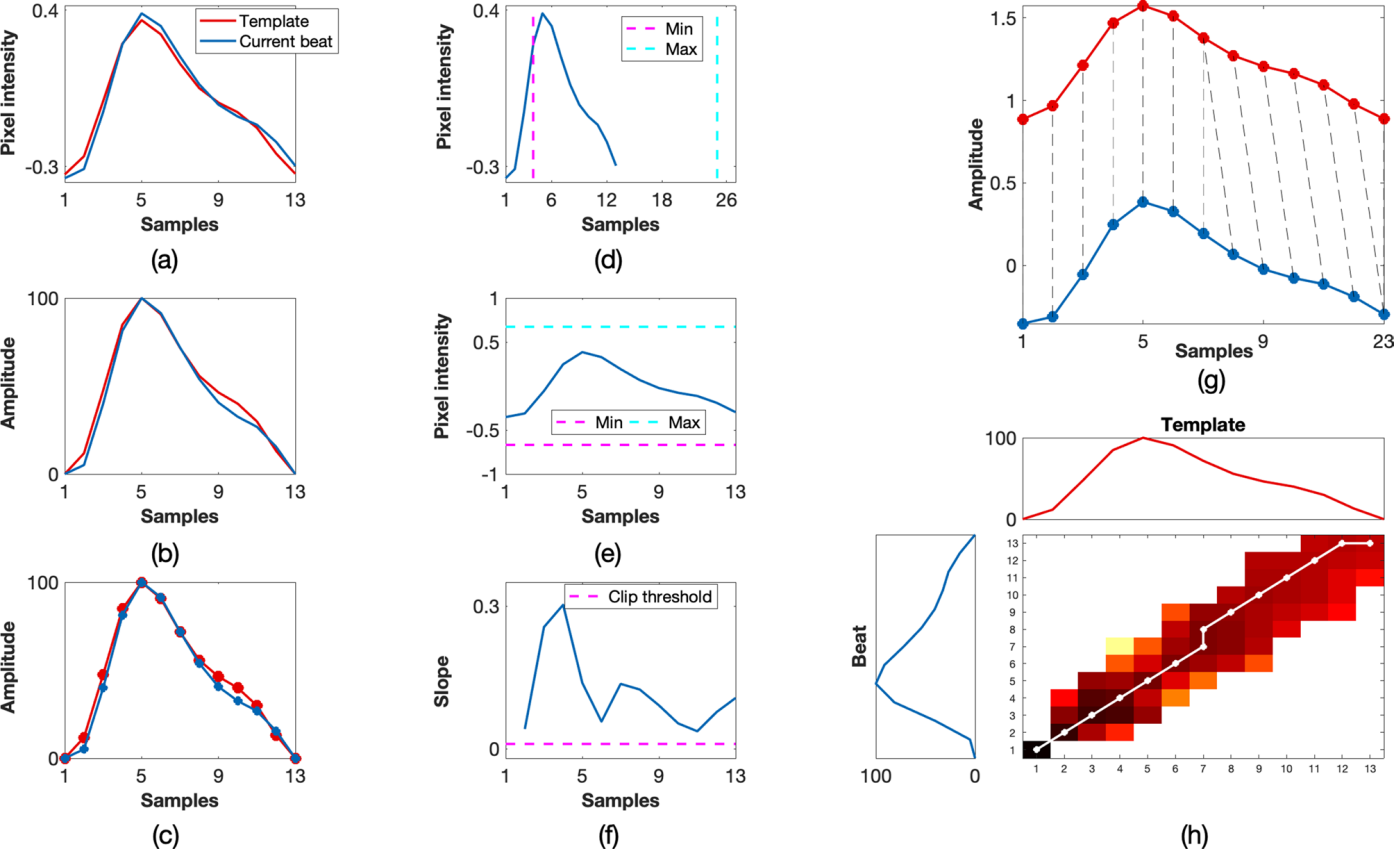
Example of a good-quality beat. The resultant combined $SQI_{\mathrm{beat}}$ value was 1. (**a**) Input template and beat, (**b**) rotated and normalised template and beat, (**c**) piece-wise linear approximation, (**d**) check if the beat was within physiological bounds for heart rate, (**e**) check if the beat was within the amplitude thresholds for the current window, (**f**) clipping detection, (**g**) DTW showing the minimum path with a cost of 3.58, (**h**) accumulated cost matrix showing the minimum path.

If any of the binary quality indices were flagged as invalid, the beat was rejected and no further processing was carried out. Otherwise, the quality of the beat was determined by the minimum DTW distance in Eq. (17). For good-quality beats, the DWT distance between the given PPGi beat and its corresponding window was low, resulting in $SQI_{\mathrm{dtw}}$ values that were typically greater than 0.80. On the contrary, poor-quality beats had much higher *DTW* distances and corresponding lower $SQI_{\mathrm{dtw}}$ values. Figure 10 shows an example of a poor-quality beat, whereas Fig. 11 shows how the metrics were used to identify a good-quality beat.

#### Computing heart rate for each ROI

Each waveform extracted for each of the three colour channels from all of the 81 $ROI_S$ was subdivided into 15-s running windows with a separation between consecutive windows of 1 s. Longer windows can lead to additional delays in a real-time implementation and decrease the percentage of data for which heart rate can be estimated as patient activity can cause more windows to be rejected. Therefore, heart rate estimates were reported every second.

The heart rate for each window from every input PPGi signal was computed individually using three algorithms: beat counting, Fast Fourier Transform (FFT) and a method based on an Autoregressive (AR) model. The SQI for each heart rate estimate for the three methods was taken as the mean of the beat-by-beat SQI for each pulse within the window for each $ROI_S$:

19 $$\begin{aligned} \begin{aligned} SQI_{{ROI_{S,i}}} = mean( SQI_{\mathrm{beat}}( b_k ) ) \quad&, \quad \forall \> b_k \in ROI_{S,i} \\ \quad&, \quad i = \{1,2,\ldots ,81\} \end{aligned} \end{aligned}$$

For the first method, once the beat onsets and peaks were located and the quality of the signals was computed, the heart rate estimate for a given window was taken as the number of pulses divided by the time interval from the first onset to the last onset. For the FFT method, the frequency of the maximum peak in the spectrum was taken to be the heart rate value for the window. A Hamming window was applied to the input PPGi window to minimise the effect of spectral leakage.

An Autoregressive model is a type of random process that attempts to predict the output of a system based on its previous outputs. It can be specified for a sample time series $x_t$ as:

20 $$\begin{aligned} x_t = c + \displaystyle \sum _{i=1}^p a_i x_{t-i} + \varepsilon _t \end{aligned}$$

where *c* is a constant (often omitted for simplicity), *p* is the model order, $a_1,\ldots ,a_p$ are the model parameter coefficients and $\varepsilon _t$ is a white noise Gaussian process. The current value of $x_t$, is a linear combination of the *p* previous values of $x_t$ and the current value of $\varepsilon _t$. The transfer function in the Z-domain relating the input to the output is given by:

21 $$\begin{aligned} H(Z) = \frac{1}{\displaystyle \sum \nolimits _{k=1}^p a_k Z^{-k}} = \frac{Z^p}{(Z-Z_1)\ldots (Z-Z_p)} = \frac{Z^p}{\mathbf {P}_1 \mathbf {P}_2\ldots \mathbf {P}_p} \end{aligned}$$

The denominator in Eq. (21) can be factored into *p* terms, where $\mathbf {P}_1 \mathbf {P}_2\ldots \mathbf {P}_p$ are vectors extending from any point *Z* in the complex plane to each of the *p* poles $Z_1,Z_2,\ldots ,Z_p$ of *H*(*Z*). As *H*(*Z*) has no finite zeros, AR can be viewed as the output of an all-pole infinite impulse response filter whose input is white noise.

After computing the Autoregressive model for the PPGi window, the pole with the highest magnitude (largest radius in the zero-pole diagram) was identified and its frequency (angle on the pole-zero diagram) was used as the heart rate estimate.

#### Pole cancellation (ARk)

A challenge during video recordings in a real hospital setting was the ambient light interference from artificial light sources^33^. The modulation of fluorescent lamps powered by alternating current is well known^34^, resulting in flicker at twice the frequency of electrical supply (50 Hz in Europe results in a flicker at 100 Hz). The 100 Hz frequency of intensity variation, was aliased down to much lower frequencies because of the camera frame rate (15 FPS). Aliasing components (artefacts) were often found at frequencies such as 4 Hz or 2 Hz. However, it was not possible to predict the exact frequencies from this aliasing process. It was not effective simply to filter at specific frequencies as the filters would have needed to be re-tuned in each setting to track the aliasing artefacts.

**Figure 12:**
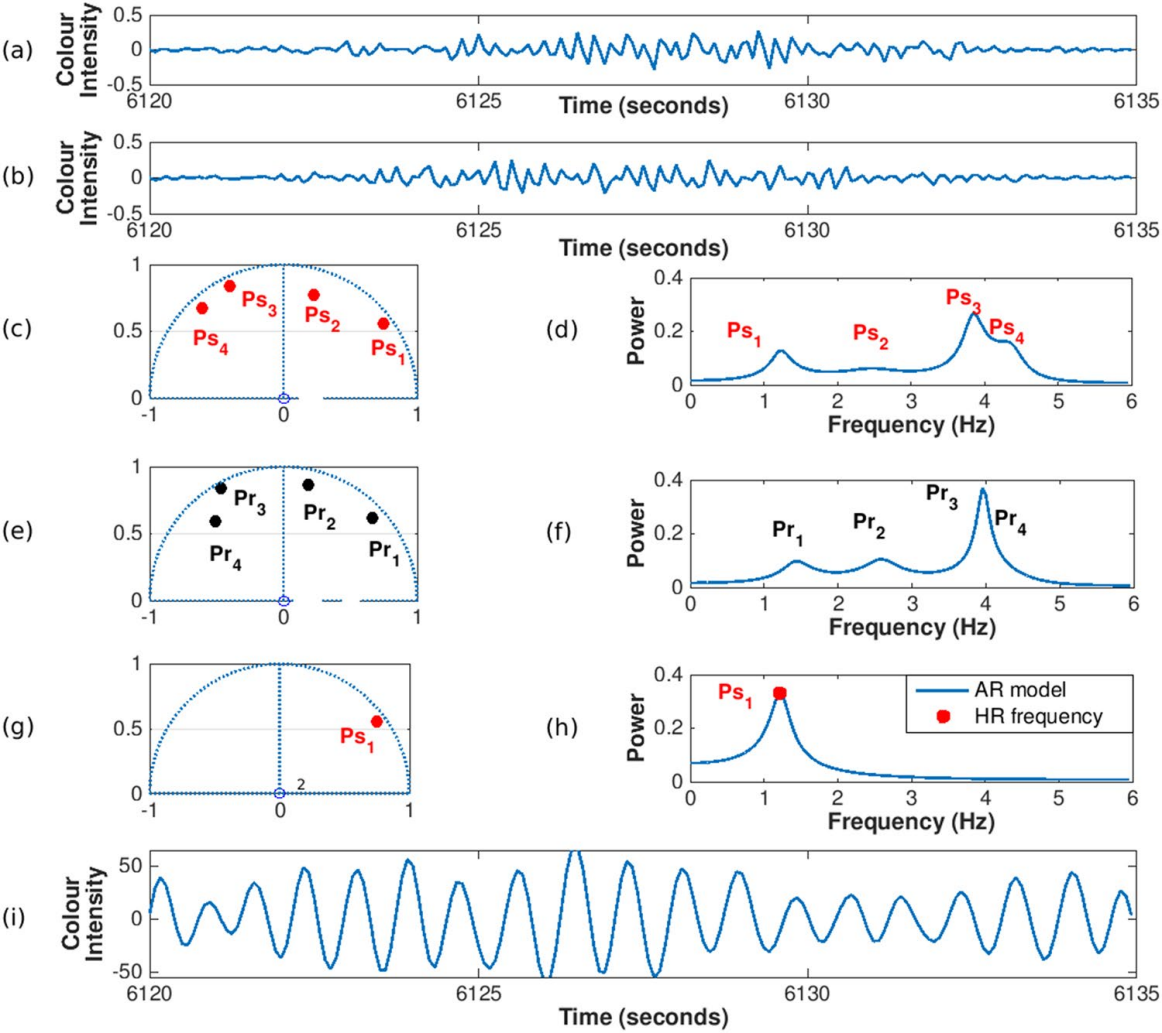
AR pole cancellation process for a 15-s window from the (**a**) $ROI_S$ and (**b**) $ROI_R$. (**c**) Zero-pole plot and (**d**) frequency components for $ROI_S$. (**e**) Zero-pole plot and (**f**) frequency components for $ROI_R$. (**g**) Similar poles from $ROI_R$ in $ROI_S$ are cancelled creating a new autoregressive model ARk and (**h**) the power spectrum is computed from the remaining pole. The heart rate estimate is taken as the peak of the reconstructed power spectrum at 1.2 Hz, corresponding to a heart rate of approximately 72 beats/min. (**i**) From the remaining pole, a signal can be generated from a white noise input source.

To reduce the ambient light interference, a reference signal was extracted from an area on the background wall, labelled $ROI_R$ (see Fig. 6b). Because the signals extracted from the skin areas and background were both imaged by the same camera at the same time intervals, aliasing artefacts were present in both $ROI_R$ and each $ROI_S$. However, the signals extracted from each $ROI_S$ additionally had spectral components corresponding to the patient’s heart rate. As with the previous three methods, all the signals from $ROI_S$ and $ROI_R$ were subdivided into 15-s windows, sliding each window by 1 s. An AR model of order 8 was computed for both $ROI_S$ and $ROI_R$ for each window. The non-physiological frequency components were identified as the poles which were common to both the AR models. The non-physiological frequency components in $ROI_S$ were subtracted from $ROI_R$ by cancelling them from the denominator in Eq. (21) for the AR model for $ROI_S$. Poles were regarded as present in both the face and background wall if they were within two angular degrees of each other. A new transfer function $H'(z)$ was computed without these cancelled poles in the denominator, representing the transfer function for the light intensity from the patient’s face without the ambient light interference. An example of the pole cancellation process is shown in Fig. 12 for a 15-s window. After the non-physiological frequency components have been cancelled, the peak in the reconstructed power spectrum from the new AR model was computed and its frequency was used as the heart rate estimate for the window.

#### Data fusion

Once the individual heart rate estimates had been computed for each colour channel from each of the 81 $ROI_S$, the overall heart rate estimate for the patient was computed by combining each of the four methods described in the previous sections using three data fusion techniques.

**Median**: The first data fusion method computed the heart rate estimate for a given window *w*, labelled $HR_w$, as simply the median of all the heart rate estimates from each colour channel from the $ROI_S$ that had an associated SQI greater than 0.8, as defined by the following equation:

22 $$\begin{aligned} \begin{aligned} HR_w = median(HR_{ROI_{S,i}}) \quad&, \quad \forall \> SQI_{ROI_{S,i}} > 0.8 \\ \quad&, \quad i = \{1,2,\ldots ,81\} \end{aligned} \end{aligned}$$

The overall signal quality for the given window, labelled $SQI_w$, was given by the median of the SQI for each of the pulses in all the ROI within the window:

23 $$\begin{aligned} SQI_w = median(SQI_{ROI_{S,i}}) \quad , \quad i = \{1,2,\ldots ,81\} \end{aligned}$$

**Kalman 1D**: A Kalman filter was used to track the heart rate estimates computed by the *Median* method described above. We implemented a Kalman filter as a discrete-time instance of a recursive Gaussian filter^35^ which sequentially calculates an estimate for a state *x*(*k*), the heart rate, with a Gaussian probability density that evolves over time *k* according to the stochastic difference equation:

24 $$\begin{aligned} x(k) = F(k)x(k-1) + B(k)u(k) + G(k)v(k) \end{aligned}$$

where *u*(*k*) describes the control input to the state *x*(*k*). *v*(*k*) is a random variable describing the uncertainty in the process model. *F*(*k*) relates the contribution of the previous state (at time $k-1$) in the absence of process noise. *B*(*k*) and *G*(*k*) describe the contribution of the control input and noise to state transition. An observation (measurement) *z*(*k*) of the state is defined as:

25 $$\begin{aligned} z(k) = H(k)x(k) + D(k)w(k) \end{aligned}$$

where *w*(*k*) is a random variable describing the uncertainty of the observation. *H*(*k*) and *D*(*k*) describe the contribution of state and noise to the observation *z*(*k*).

An important assumption for a Kalman filter is that the random variables *v*(*k*) and *w*(*k*), representing the process and measurement noise respectively, are Gaussian distributed, uncorrelated, with zero-mean and known covariances *Q* and *R*:

26 $$\begin{aligned} P(v) \sim \mathcal {N}(0, Q) \quad , \quad P(w) \sim \mathcal {N}(0, R) \end{aligned}$$

We define $\hat{x}(k|k-1)$ as the *a priori* state estimate, or prediction, at a time *k* given only information up to time $k-1$. After an observation *z*(*k*) is made at time *k*, we can compute the *a posteriori* state estimate $\hat{x}(k|k)$ as a linear combination of the *a priori* estimate and a weighted difference between the actual measurement *z*(*k*) and the measurement prediction $H(k)\hat{x}(k|k-1)$:

27 $$\begin{aligned} \hat{x}(k|k) = \hat{x}(k|k-1) + \mathbb {K}[z(k) - H(k)\hat{x}(k|k-1)] \end{aligned}$$

The term $[z(k) - H(k)\hat{x}(k|k-1)]$ is often referred as the innovation or measurement residual. It is an important measure of the deviation between the predictions and the observation sequence. $\mathbb {K}$ is the Kalman gain, defined as:

28 $$\begin{aligned} \mathbb {K}(k) = \frac{ P(k|k-1) H^\intercal (k) }{ H(k)P(k|k-1)H^\intercal (k) + R(k)} \end{aligned}$$

where $P(k|k-1)$ is the *a priori* estimate error covariance at a time *k* given only information up to time $k-1$. The Kalman gain $\mathbb {K}$ controls the relative weight between the *a priori* state estimate and the actual measurement. It is usually tuned to achieve a particular performance or to model the underlying behaviour of the state to track.

Given that a single state (the heart rate estimate) needs to be tracked, and following Tarassenko et al.^36^ and Li et al.^28^, the Kalman filter was reduced according to the following conditions: No control input was assumed, therefore $$u(k) = 0$$. The heart rate at time *k* was assumed to be equal to the heart rate at time $$k-1$$, therefore $$F(k) = 1 $$. However, it is known that heart rate varies from beat to beat, a phenomenon referred as heart rate variability. This was modelled by assigning a value of 0.1 to the process noise variance *Q* for time periods during which the patient was quiet. When the patient was moving, the heart rate was expected to change. This was modelled by assigning a value of 0.2 to the process noise variance *Q* during periods of motion, identified by the activity index described in the video processing section. Given that the process noise was modelled using the two different values of *Q*, a value of 1 was assigned to *G*(*k*). A value of 1 was also assigned to *H*(*k*) and *D*(*k*). Therefore, the *a posteriori* state estimate was simplified to:

29 $$\begin{aligned} \hat{x}(k|k) = \hat{x}(k|k-1) + \mathbb {K}[z(k) - \hat{x}(k|k-1)] \end{aligned}$$

and the Kalman gain to:

30 $$\begin{aligned} \mathbb {K}(k) = \frac{ P(k|k-1) }{ P(k|k-1) + R(k)} \end{aligned}$$

In our proposed algorithm, the Kalman gain is mainly controlled by the measurement noise covariance *R*(*k*). Similarly to^28^, in order to assign a higher weight to heart rate estimates computed from good-quality PPGi signals, the measurement error covariance was modified as:

31 $$\begin{aligned} R(k) = R(k) * exp( \frac{1}{SQI_w^2} -1) \end{aligned}$$

where $SQI_w$ is the signal quality index computed for the heart rate estimate for the given data window. Adjusting the estimates based on the SQI had the effect that when the signal quality was low ($SQI_w \rightarrow 0$), the Kalman filter prediction $\hat{x}(k|k-1)$ was trusted more over the measurement *z*(*k*):

32 $$\begin{aligned} \lim _{SQI_w \rightarrow 0} R(k) * exp( \frac{1}{SQI_w^2} -1) = \infty \quad \Longrightarrow \quad \lim _{R(k) \rightarrow \infty } \frac{ P(k|k-1) }{ P(k|k-1) + R(k)} = 0 \quad \Longrightarrow \quad \hat{x}(k|k) = \hat{x}(k|k-1) \end{aligned}$$

Conversely, when the quality of the data window was high ($SQI_w \rightarrow 1$), the measurement error covariance was unaffected. This had the effect that the measurement residual was assigned a higher weight in Eq. (29). Therefore, the actual measurement *z*(*k*) was trusted over the prediction to compute the *a posteriori* state estimate $\hat{x}(k|k)$. Specifically:

33 $$\begin{aligned} \lim _{SQI_w \rightarrow 1} R(k) * exp( \frac{1}{SQI_w^2} -1) = R(k) \end{aligned}$$

**Kalman ND**: Each colour channel from every ROI was tracked with an individual Kalman filter, producing one estimate per ROI and per colour channel. The output from each Kalman filter was then combined following the proposed methods of Tarassenko et al.^36^ and Li et al.^28^. This method is called “Kalman ND”. The overall heart rate estimate for the current window $HR_w$ is therefore defined as:

34 $$\begin{aligned} HR_w = \sum _{l=1}^{n} \left( \frac{ \prod _{j=1,j \ne l}^n \sigma _j^2 }{ \sum _{i=1}^{n}( \prod _{j=1,j \ne i}^n \sigma _j^2 ) } * HR_{ROI_{S,l} } \right) \> , \> l = 1,2, \ldots ,n \end{aligned}$$

where $HR_{ROI_{S,l}}$ represents the heart rate estimate for the ROI *l*. The residual $\sigma$ is weighted by the SQI and is given by:

35 $$\begin{aligned} \sigma _i^2 = \left( \frac{r_i}{SQI_i} \right) ^2 \end{aligned}$$

where $r_i$ is the Kalman filter residual for the ROI *i* and $SQI_i$ is the signal quality index for the data window for the ROI *i*.

### Respiratory rate estimation

**Figure 13:**
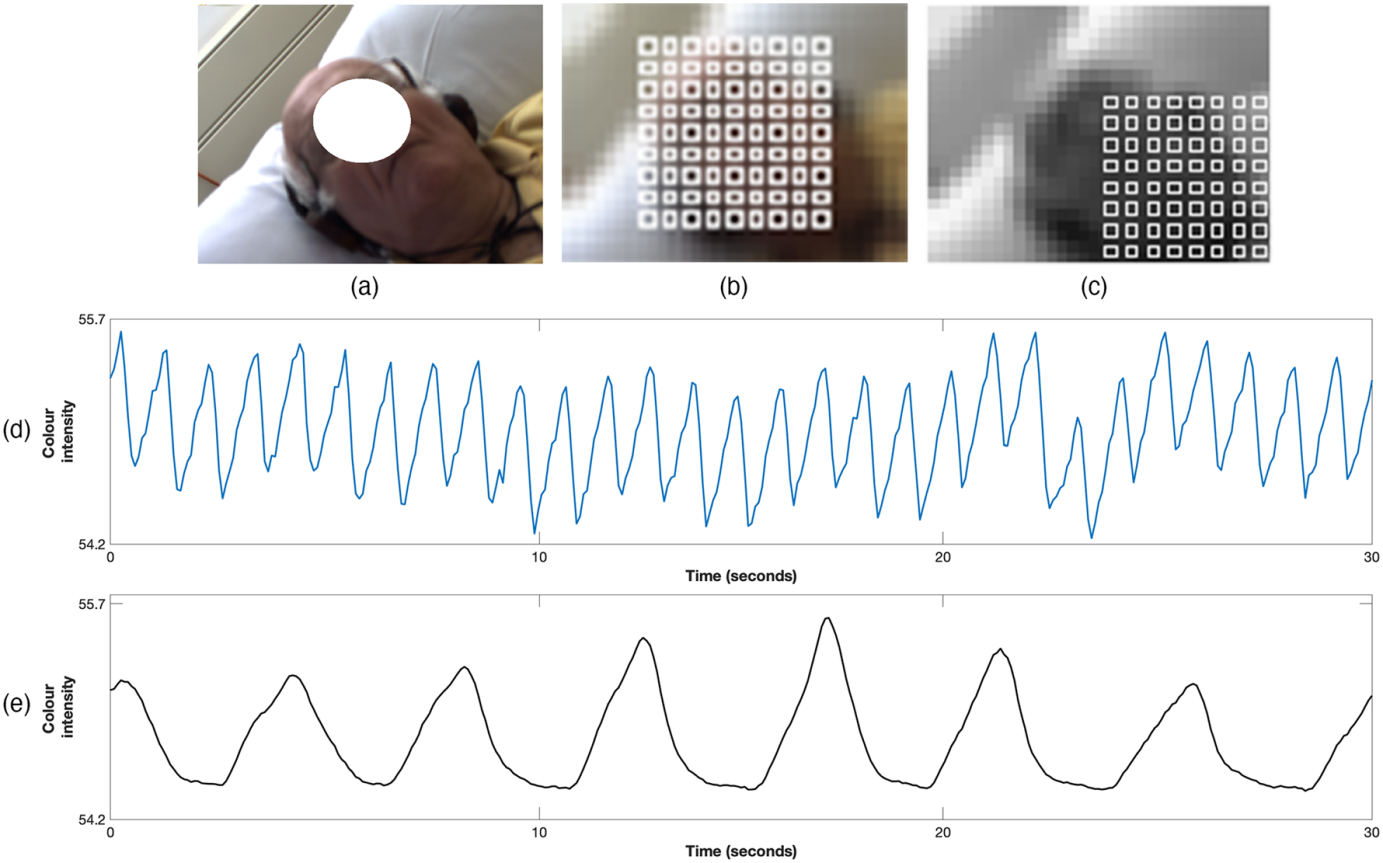
ROI selection for respiratory rate estimation for (**a**) one sample image frame. Several ROI were defined for two main areas: (**b**) skin areas across the patient’s face defined on colour images, (**c**) the patient’s upper torso defined on grey-scale images. 30-s pixel intensity time series extracted from (**d**) the green channel from a ROI on the patient’s face, (**e**) grey-scale images from a ROI on the patient’s upper chest.

Once stable periods were identified from the video recordings, respiratory signals were extracted from multiple ROI’s located on two main areas: colour images from skin areas across the patient’s face (see Fig. 13a,b) and grey-scale images from the patient’s upper torso (see Fig. 13c). The nature of these two types of signals differ in principle. Although the signals extracted from the patient’s skin were pulsatile time series in synchrony with the cardiac pulse, they were also modulated by respiration (Fig. 13d). The signals extracted from the upper torso contained respiratory-induced motion patterns, principally from regions around the thorax and diaphragm (Fig. 13e). Respiratory rate was then computed by using data fusion algorithms from the analysis of the signals extracted from all ROI’s.

#### Respiratory signal extraction from the face

The signals extracted from ROI on the patient’s face, correspond to the PPGi time series extracted and analysed in the previous sections for heart rate estimation. Similarly to the PPG waveform recorded by a pulse oximeter, there is an amplitude modulation due to respiration in the PPGi signals extracted from the video data. Therefore, respiratory signals were extracted from each PPGi waveform following the Respiratory-Induced Amplitude Variation (RIAV) algorithm^37^. Each PPGi signal extracted from ROI across the patient’s face was subdivided into 30-s windows with an overlap of 5 s between them. The following sections describe the signal processing methods applied to each individual waveform, analysing the PPGi data stream for each ROI independently.

The peaks and onsets were detected following the adaptive Boxed Sum Slope Function (uBSSF) algorithm previously described in this paper. Subsequently, a respiratory waveform was extracted by computing the amplitude of the PPGi signal, defined as the difference between the peaks and onsets for every PPGi pulse. The resulting waveform was then resampled to 4 Hz.

The number of respiration signals computed from every PPGi signal extracted from ROI’s on a patient’s face is large, up to 243 PPGi waveforms for the three colour channels from each of the 81 regions (9x9 grid). As the breathing pattern of a patient is global, the data from all these regions of interest potentially contain similar respiratory frequency information. To reduce the number of signals to process, Principal Component Analysis (PCA) was applied to all the respiration signals computed from every PPGi signal. The first five components that contain frequency content in the valid respiratory range, taken to be between 6 to 42 breaths/min, were selected for further processing.

#### Respiratory signal extraction from the upper torso

As the patient’s upper torso was covered by clothing, the signals extracted did not depend on colour changes due to blood flow, but rather depended on the subject’s motion patterns. Each colour image frame from the video data was converted to a grey-scale version using the following equation:

36 $$\begin{aligned} Gray_{i,j} = 0.2989 * R_{i,j} + 0.5870 * G_{i,j} + 0.1140 * B_{i,j} \end{aligned}$$

where R,G and B are the colour intensity for the red, green and blue pixels respectively, located at the row *i* and column *j* across the image frame. The mean over each region of interest was subsequently computed, as shown in Fig. 13e.

As with the signals extracted from the patient’s face, PCA was applied to all the respiration signals computed from every ROI on the patient’s chest. The first five components that contain frequency content in the valid respiratory range, taken to be between 6 to 42 breaths/min, were selected for further processing.

#### Signal quality assessment

In ideal conditions, the respiration signal is a sinusoidal waveform with a period corresponding to the respiratory rate of the patient. Therefore, the extent to which the signal can be described by a single frequency is a measure of its quality. Barlow^38,39^ proposed the use of Spectral Purity Index (SPI) as a heuristic parameter designed to reflect the bandwidth of a signal, defined by the following equation:

37 $$\begin{aligned} \Gamma _{SPI}(n) = \frac{ \bar{w}^2_2(n) }{\bar{w}_0(n) \bar{w}_4(n)} \end{aligned}$$

where $\bar{w}_i$ is the $i^{th}$ order spectral moment defined by:

38 $$\begin{aligned} \bar{w}_i = \int _{-\pi }^{\pi } \omega ^i S_x(e^{j\omega }) d\omega \end{aligned}$$

where $S_x(e^{j\omega })$ is the power spectrum of the signal as a function of angular frequency $\omega =2\pi f$, *f* being the frequency of the signal.

SPI tends to unity for a noise-free sinusoidal signal. It decreases to zero as the bandwidth of the signal increases. Some PCA components included sources other than respiration. To choose which PCA component to select for respiratory rate estimation, a decision rule based on its quality was applied. The quality index for the *i*th PCA component over a 30-s time window *k* was defined as:

39 $$\begin{aligned} SQI_{(k_i)} = {\left\{ \begin{array}{ll} \Gamma _{SPI}(k_i) &{} , \text {if } (0.1\,\hbox {Hz} \le FFT_{peak}(k_i) \le 0.7\,\hbox {Hz}) \text { and } (PCA_{ex}(k_i)> 0.3) \text { and } (SNR(k_i) > 1) \\ 0 &{} , otherwise \end{array}\right. } \end{aligned}$$

where $\Gamma _{SPI}(k_i)$ is the “spectral purity index” as defined by Eq. (37), $FFT_{peak}(k_i)$ is the dominant frequency for the window (as determined by the peak of the FFT), $PCA_{ex}(k_i)$ is the fraction of the total variance explained by the PCA component and $SNR(k_i)$ is the signal-to-noise ratio. When the peak of the FFT was within the physiological range for respiratory rate (determined to be from 0.1 Hz to 0.7 Hz, corresponding to a range from 6 breaths/min to 42 breaths/min), the PCA component explained more than $30\%$ of the total variance and the signal-to-noise ratio was greater than 1.0, then the SQI for the given PCA component and window was equal to the SPI. Otherwise, the SQI was equal to zero and the PCA component was discarded.

#### Computing respiratory rate

To estimate respiratory rate from the extracted respiratory signals, the input waveforms were subdivided in 30-s windows. Longer windows could lead to additional delays in a real-time implementation and decreased the percentage of data to be estimated as patient activity could cause more windows to be rejected. The separation between consecutive windows was chosen to be 5 s to provide a large amount of overlap between windows. Hence, respiratory rate estimates are reported every 5 s.

After all the respiratory signals were extracted, respiratory rate was estimated for each signal using an 8th-order Autoregressive Model. The “Kalman ND” algorithm described in the previous section, was applied to the respiratory signals grouped into three datasets extracted from: the patient’s face, patient’s upper torso, and all the respiratory signals combined.

## Supplementary information

Supplementary Information.

## Data Availability

The datasets generated or analysed during the current study are not publicly available due to the sensitive and identifiable nature of our data, patient consent and restrictions of the ethics protocol to protect the privacy of patients involved in the study.
