# Supplementary material for: Non-contact vital-sign monitoring of patients undergoing haemodialysis treatment
Source: Sci Rep. 2020 Oct 28;10:18529. doi: 10.1038/s41598-020-75152-z (PMC7595175; doi:10.1038/s41598-020-75152-z)
Supplement: Supplementary file 1 — Supplementary Information. [file 41598_2020_75152_MOESM1_ESM.pdf]

Non-contact vital-sign monitoring of patients undergoing haemodialysis treatment

Mauricio Villarroel[1,*], João Jorge[1], David Meredith[2], Sheera Sutherland[2], Chris Pugh[3], Lionel Tarassenko[1]

1 Institute of Biomedical Engineering, Department of Engineering Science, University of Oxford, UK
2 Oxford Kidney Unit, Oxford University Hospitals National Health Service Trust, Oxford, UK
3 Nuffield Department of Medicine, University of Oxford, UK
* mauricio.villarroel@eng.ox.ac.uk

Supplementary material

Supplementary method 1: Reference heart rate

The heart rate estimates provided by the two reference pulse oximeters used in our study (attached to the finger tip and ear lobe respectively), were computed using different proprietary algorithms and used different connection technologies to transfer the data. The pulse oximeter attached to the ear lobe was connected to the Hidalgo ambulatory device through a physical wire using a serial connection. Hidalgo provided one heart rate estimate every 5 seconds, corresponding to a data rate of 0.2 Hz. In contrast, the raw PPG signal and the heart rate estimates from the finger pulse oximeter were directly recorded and transmitted to a separate computer (which also controlled the video data acquisition) using a Bluetooth wireless connection. The finger pulse oximeter provided three heart rate estimates every second, corresponding to a data rate of 3 Hz.

According to the finger pulse oximeter manufacturer, each reported heart rate estimate was the result of averaging 8 beats, representing variable data window depending on the patient's heart rate. There was no documentation available regarding the algorithm or window size used by the ear pulse oximeter. It is important to also note that the heart rate estimates from the two pulse oximeters were reported as integers, but the rounding strategy was not documented by either of the manufacturers.

To compute estimates of SpO_2 and heart rate, pulse oximeters take advantage of the pulsatile nature of arterial blood to be identified from the absorbance effects of non-pulsatile venous blood and other body tissue. Occasionally, little shunts can open up in the blood vessels of the skin that bypass the capillaries and send arterial blood directly into the venous system. It is thought that this is part of the cooling/warming mechanism in humans, and it is likely that there are more of these shunts in the blood vessels of the earlobe than the finger[1]. This shunting of arterial blood into the veins can cause some errors in the measurements provided by a pulse oximeter[2].

Cardiovascular disease is one of the primary causes of morbidity among haemodialysis patients. Left ventricular hypertrophy (the enlargement and thickening of the walls of the heart's main pumping chamber) and arterial disease are common physiological adaptive responses to a sustained increase in cardiac work due to a prolonged haemodynamic overload[3]. It can result in an abnormal geometry of the large arterial branches and can cause the stiffening of the arterial tree. Moreover, the patients in our study were often diagnosed with other illnesses, including Diabetes (high blood glucose level), Hypertension (long-term high blood pressure), Hypovolaemia (decrease in the volume of blood in the body) and others. As such, patients were given medications that could have deteriorating side effects on their cardiovascular status and, consequently, could affect the values reported by the pulse oximeters.

Haemodialysis is an invasive treatment in itself. A surgically-formed vascular conduit (the Artereo-Venous Fistula) is placed between the venous system and the radial or brachial artery in the distal or proximal forearm, respectively. This conduit allows the clean arterial blood to bypass the capillary bed and pass directly into the venous system. The increased venous pressure and flow promote thickening or arterialisation of the vascular wall[2]. The invasive nature of the fistula at the arterial-venous shunt could also have an effect on the recordings of the two pulse oximeters used in our study.

Supplementary figure 1 shows the agreement between the original heart rate values provided by the two pulse oximeters for the entire dialysis dataset. Although no sensor bias was present in the Bland-Altman plot and the mean values cover a valid heart rate physiological range for adults, large differences exist between the measurements. As a consequence, a direct comparison (on a sample-by-sample basis) of the heart rate estimates between the video camera and each of the two pulse oximeters is potentially affected not only by physiological factors but also by the recording set-up and each of the manufacturer's processing

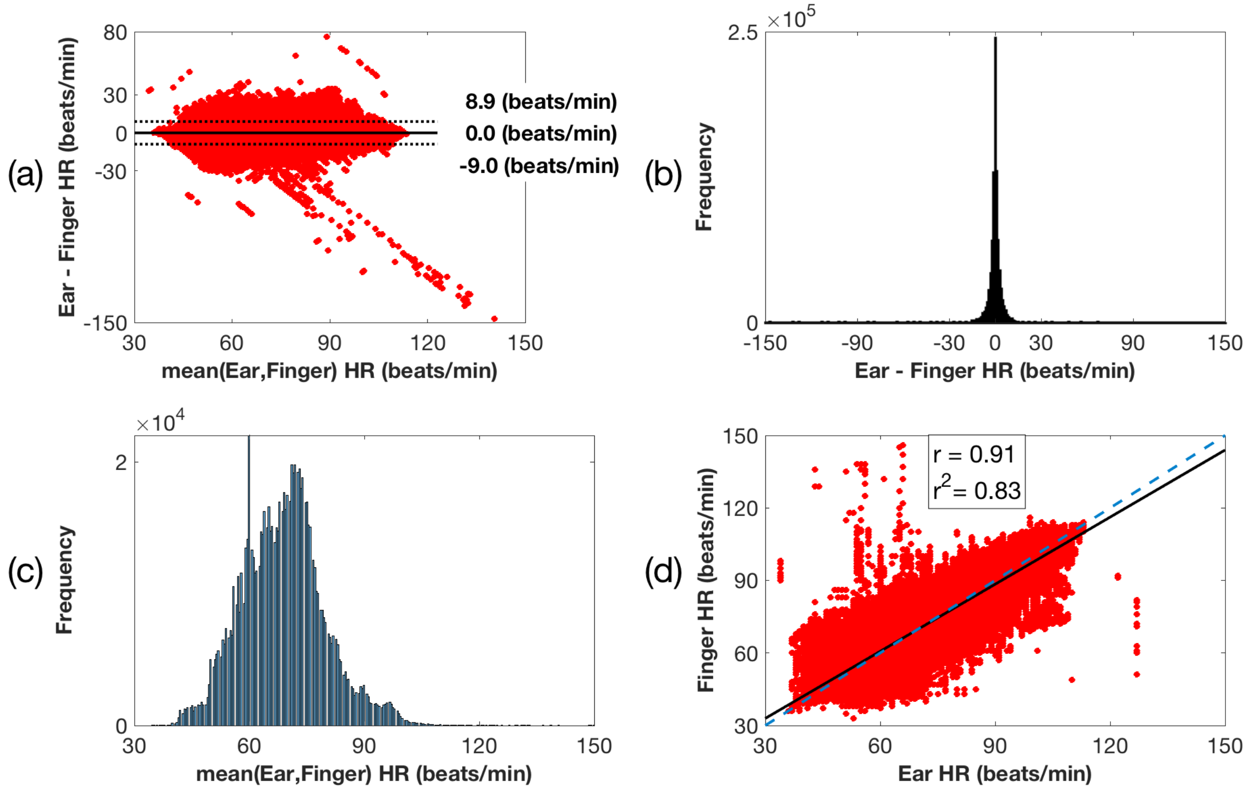

Supplementary figure 1. Agreement between the original heart rate values provided by the two pulse oximeters for the entire dialysis dataset. (a) The Bland-Altman plot presents no sensor bias but a large differences exists between the measurements. (b) The distribution of the differences between the two pulse oximeters. (c) The distribution of the mean values, spanning the heart rate physiological range for adults. (d) The correlation plot shows a high correlation between the two devices with a correlation coefficient of 0.91, even though large differences between the measurements exist.

context, leading to inaccurate performance results. Therefore, a new reference heart rate is required to be computed as the ground-truth for comparison with the estimates derived from the video camera. A more detailed description of the process of estimating the reference heart rate can be found at[4].

Computing the reference heart rate

To compute the new reference heart rate, a three-stage process is proposed: resampling, time alignment and averaging. Initially, as the two pulse oximeters provide values at different time intervals, the heart rate estimates from each of the two pulse oximeters were re-sampled to 1 Hz to match the video camera estimates reported every second. Secondly, the two signals were time aligned by subtracting the sample delay as computed from the lag for which the normalised cross-correlation has the largest absolute value for the entire recording session.

The normalised cross-correlation is a standard method of estimating the time delay between two signals. Given two time series $X = x_1, x_2, ..., x_n$ and $Y = y_1, y_2, ..., y_n$ of length N, cross-correlation measures the similarity between X and shifted (in time) copies of Y as a function of the time displacement, known as the lag. The normalised cross-correlation, at time lag t, is defined by:

$$R_{xy}(t) = \frac{E\{(x_i - \mu_X)(y_{i-t} - \mu_Y)\}}{\sigma_X \sigma_Y} = \frac{\sum_{i=1}^{N}[(x_i - \mu_X)(y_{i-t} - \mu_Y)]}{\sqrt{\sum_{i=1}^{N}(x_i - \mu_X)^2}\sqrt{\sum_{i=1}^{N}(y_{i-t} - \mu_Y)^2}} \quad (1)$$

The time delay D_{xy} of the signal Y with respect to X is computed as occurring at the maximum of the normalised cross-correlation for all possible time lags t:

$$D_{xy} = \arg\max_t |R_{xy}(t)| \quad (2)$$

Finally, the measurement of a physiological process implies some degree of error. When two sensing devices are used, neither provides an absolute correct measurement. Since the true value is not known, the mean of the two measurements is usually taken as the representative value[5]. Therefore, the new reference heart rate was computed, on a second-by-second basis, as the mean of the good-quality estimates from each pulse oximeter that do not varied by more than 5 beats/min, as recommended by a widely accepted medical standard for cardiac monitors[6].

Results

Supplementary table 1. Comparison of the heart rate estimates provided by the two reference pulse oximeters placed on the ear lobe and the finger for the overall patient population, comprising a total recording length of 304.1 hrs.

Data	MAE (beats/min)	MAD (beats/min)	Time (%)
Original values	27.5	94.0	304.1 (100%)
Good quality values	2.5	3.8	263.5 (86.6%)
New reference HR	1.2	1.3	242.7 (79.7%)

In our dataset, there were originally 104 dialysis video sessions recorded from 40 patients, corresponding to a total recording time of 369.1 hrs. For 12 sessions, the recording from at least one pulse oximeter was interrupted due to patient discomfort, medical intervention, equipment malfunction or other external factors. 8 sessions were discarded because the video camera recording equipment was interrupted by the clinical staff or the recording was not completed. Therefore, there were simultaneous recordings with both pulse oximeters and camera for only 84 sessions, corresponding to 304.1 hrs.

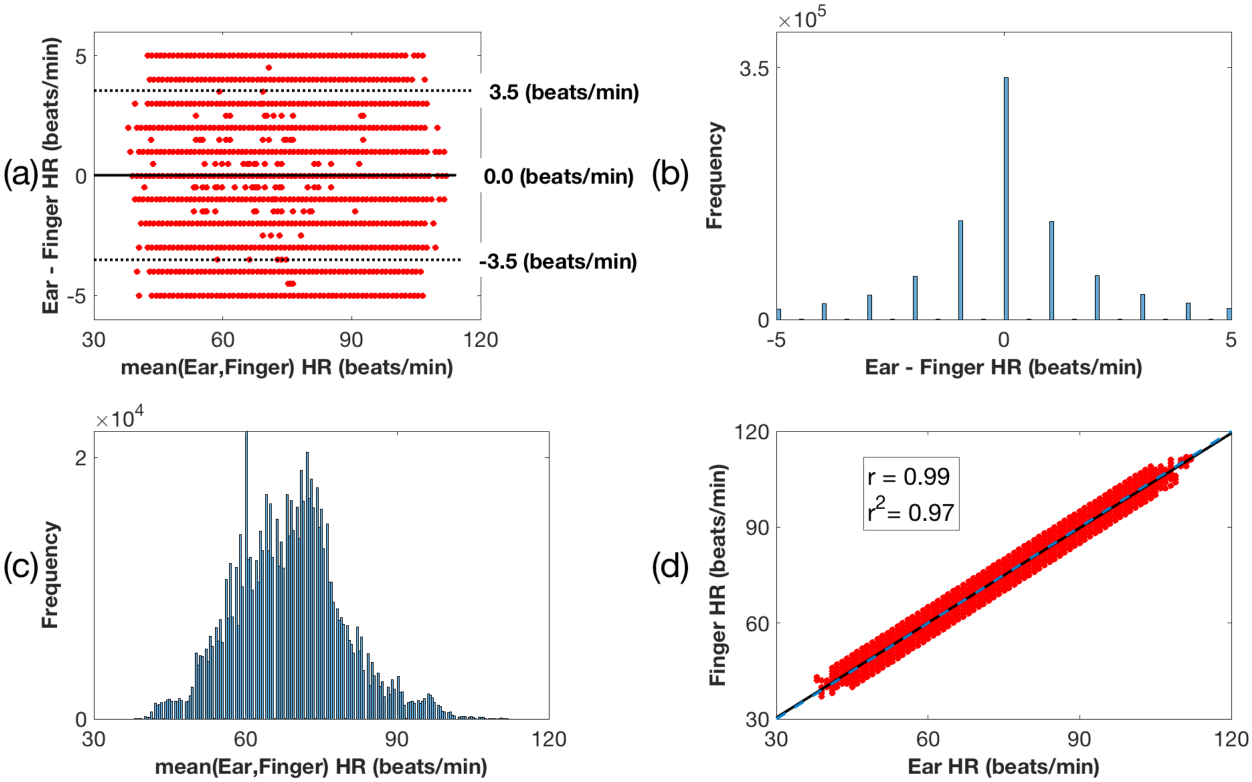

Supplementary figure 2. Agreement between the heart rate values provided by the two pulse oximeters used to compute the reference heart rate for the entire dialysis dataset, comprising a total recording time of approximately 304.1 hrs. (a) The Bland-Altman plot presents no sensor bias. (b) The distribution of the difference between the two pulse oximeters is normally distributed. (c) The distribution of the mean values shows that most of the heart rate estimates are within the expected physiological range. (d) The plot shows a strong correlation between the two devices with a correlation coefficient of 0.99.

The original values provided by the manufacturers had a large MAE of 27.5 beats/min with a MAD of 94.0 beats/min (see supplementary table 1). When comparing only the values reported as good-quality estimates (not flagged as errors by the manufacturer), the error reduced to a MAE of 2.5 beats/min and a MAD of 3.8 beats/min for approximately 263.5 hrs (86.6%). Finally, the resulting new reference heart rate had a MAE of 1.2 beats/min and a MAD of 1.3 beats/min for approximately 242.7 hrs (79.7%).

Supplementary figure 2 shows in more detail the agreement of the heart rate estimates from the two pulse oximeters for the values used in computing the reference heart rate. The Bland-Altman plot presents no sensor bias with narrow differences across the heart rate physiological range, implying a good agreement between the two measurements. The distribution of the differences is normally distributed. The mean values are within the expected physiological range for adults. The values from the two devices are now highly correlated with a positive correlation coefficient of 0.99.

Supplementary method 2: Reference respiratory rate

The Hidalgo's Equivital LifeMonitor is a wearable ambulatory monitoring system for collecting multiple physiological parameters. It provides, amongst other signals, two simultaneous respiratory rate estimates, the first one computed from the thoracic chest belt, and the second using the ECG. The two respiratory rate estimates were computed by the manufacturer every 5 seconds.

The chest belt is a harness that the patient wears on the upper torso. It contains an elastic thoracic expansion band that measures the patient's chest wall expansion and contraction as the patient breathes in and out. From the analysis of these movements, it derives a respiration signal at a sampling rate of 25.6 Hz. The ECG electrodes are housed inside the same fabric as the thoracic expansion band. The device records two channels of ECG at a sampling rate of 256 Hz.

The chest belt features a system of sensors embedded in a wearable textile fabric manufactured in a limited number of sizes to be able to cover most patients' body sizes. To record high-quality data, the belt has to be tightly fitted around the patient's torso, positioned in line with the bottom of the pectoral muscles. A shoulder strap provides additional support and improves mobility. Therefore, the quality of the signals recorded from the device, and hence the computed respiratory estimates, relies on the proper fit of the chest harness.

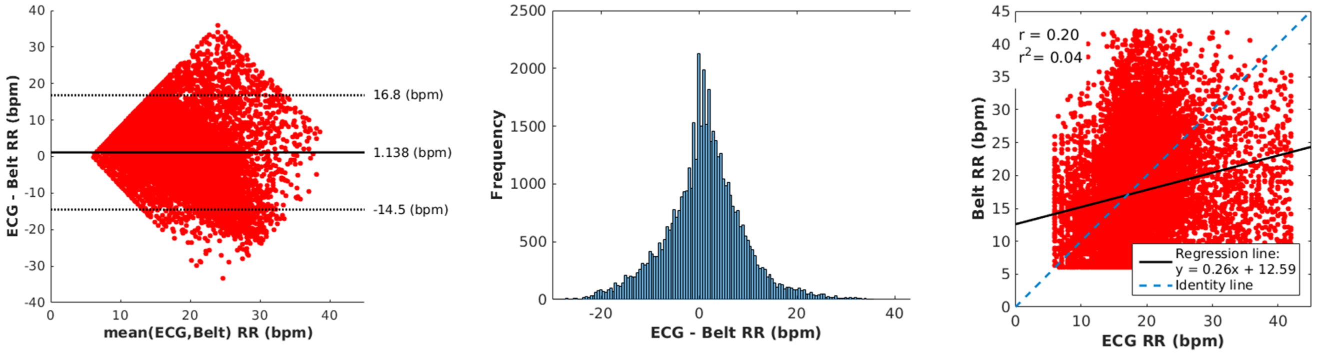

(a) (b) (c)

Supplementary figure 3. The agreement between the respiratory rate estimates provided by Hidalgo's LifeMonitor using the ECG and chest belt waveforms for the overall data set when the estimated values are limited to the physiological range of 6 - 42 breaths/min. (a) The Bland-Altman plot presents a positive bias of 1.14 breaths/min. (b) The distribution of differences between the estimated values and the reference. (c) The correlation plot shows no correlation between the estimates, with a correlation coefficient of 0.20.

When comparing the respiratory rate estimates provided by the manufacturer, the mean absolute error was 26.4 breaths/min with a mean absolute deviation of 23.9 breaths/min. Most of the errors originated from extreme values outside a patient's physiological range. This large discrepancy can be partly explained by the regular activity of the patient during the haemodialysis session or can be related to inaccuracies in the recording equipment. Even when limiting the respiratory rates estimates to a physiological range between 6 to 42 breaths per minute, the errors are still large. The MAE decreased to 6.0 breaths/min with a MAD of 5.4 breaths/min. Although the error decreases, the correlation coefficient is still low at 0.20, showing no significant correlation (see supplementary figure 3).

Given the errors presented above, the respiratory rate estimates provided by the Hidalgo LifeMonitor are not suitable as reference values to be compared with the respiratory rate estimates computed from the video camera. Therefore, new gold standard reference values need to be computed using state-of-the-art methods for analysing and fusing the two source signals: the ECG and the chest belt. A more detailed description of the process of estimating the reference respiratory rate can be found at[4].

Computing the reference respiratory rate

From the two main source signals, the ECG and the chest belt, the latter already recorded a respiration signal from which a respiratory rate could be computed directly. Therefore, the process started with the extraction of respiratory signals from the ECG.

The signal quality of the reference data was not provided by the manufacturer. Well known algorithms, commonly used in the research community and validated against publicly available physiological databases, were used to detect and assess the quality of each ECG beat. Subsequently, three respiratory signals were extracted from the ECG: ECG-derived respiration (EDR), respiratory sinus arrhythmia (RSA) and R-peak amplitude (RPA).

Once the four respiratory signals were extracted (one from the chest belt and three from the ECG), each signal was pre-processed following four steps: Firstly, a band-pass filter was applied to reduce the effects of frequency content outside the respiratory range. Secondly, the location of each breath peak was computed using a peak and valley algorithm. Thirdly, the quality of the respiration signal was assessed to discard periods for which the signal was noisy. Finally, respiratory rate was estimated from each signal over a 30-second sliding window with a step size of 5 seconds using two methods: a time-domain technique by simply counting the number of breaths over the 30-second window, and a frequency-domain technique based on autoregressive models.

Supplementary table 2. Comparison of the respiratory rate estimates provided by the two reference pulse oximeters placed on the ear lobe and the finger for the overall patient population, comprising a total recording length of 304.1 hrs.

Data	MAE (beats/min)	MAD (beats/min)	Time (%)
Original values	26.4	23.9	304.1 (100%)
Valid range	6.0	5.4	94.3 (31%)
New reference RR	0.6	0.5	101.4 (32.3%)

Two new respiratory rate estimates were computed, one for each method, by combining the individual respiratory rates from each individual signal using a data fusion algorithm. Finally, the new reference respiratory rate was computed as the mean of the combined respiratory rate estimates from both methods during which the data were of good quality and their difference was less than 2 breaths/min.

Results

Supplementary table 2 compares the original respiratory rate estimates from the ECG and chest belt with the new reference respiratory rate for the entire dataset when video was recorded. From 304.1 hours, 32.3% (101.4 hours) of the estimates were within 2 breaths/min, with a mean absolute error of 0.6 breaths/min and a mean absolute deviation of 0.5 breaths/min.

Supplementary figure 4 shows a positive correlation of 0.96 with minimum bias in the Bland-Altman plot of 0.1 breaths/min. The distribution of differences between the two estimates, shown in (c) is normally distributed.

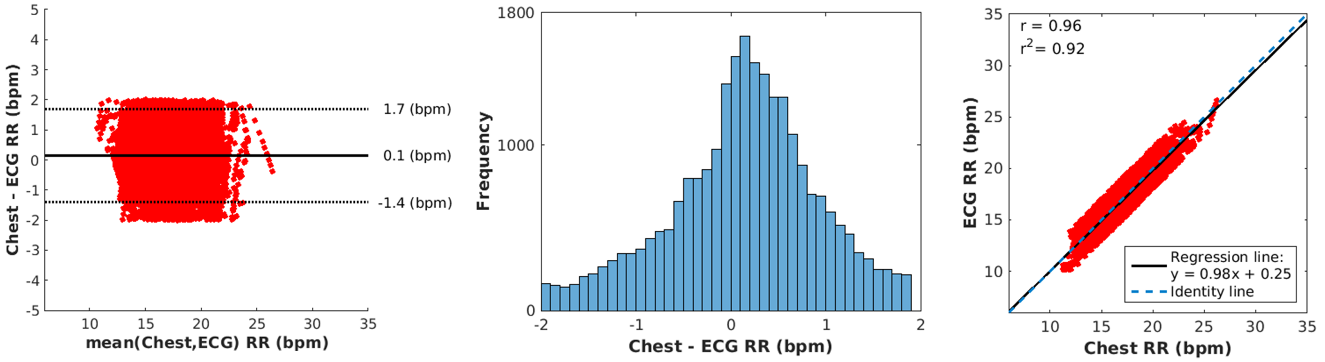

(a) (b) (c)

Supplementary figure 4. The agreement between the respiratory rate estimates computed from the ECG and chest belt for the overall data set (304.1 hours) when the difference between the estimated values is limited to 2 breaths/minute. (a) The Bland-Altman plot presents a bias close to zero. (b) The distribution of the differences between the estimated values and the reference. (c) The correlation plot shows a strong positive correlation between the estimates, with a correlation coefficient of 0.96.

The new re-derived respiratory rate estimates are consistent with values in the published literature. They were found to be of poor quality for between 37% to 61% of the time[7,8]. These new gold standard estimates will now be used for comparison with the respiratory rate estimates from the video camera described in the next chapter.

Supplementary references

1. Charkoudian, N. Skin blood flow in adult human thermoregulation: how it works, when it does not, and why. In *Mayo Clinic Proceedings*, vol. 78, 603–612 (Elsevier, 2003).
2. Meredith, D. *Continuous Monitoring During Haemodialysis*. Ph.D. thesis, University of Oxford (2014).
3. Meeus, F. *et al.* Pathophysiology of cardiovascular disease in hemodialysis patients. *Kidney international* **58**, S140–S147 (2000).
4. Villarroel, M. *Non-contact vital sign monitoring in the clinic*. DPhil thesis, University of Oxford (2017).
5. Bland, J. M. & Altman, D. G. Statistical methods for assessing agreement between two methods of clinical measurement. *Int. J. Nurs. Stud.* **47**, 931–936 (2010).
6. ANSI. Cardiac monitors, heart rate meters, and alarms. Standard ANSI/AAMI EC13: 2002, American National Standards Institute - Association for the Advancement of Medical Instrumentation (2002).
7. Cecil, W. T., Thorpe, K. J., Fibuch, E. E. & Tuohy, G. F. A clinical evaluation of the accuracy of the Nellcor N-100 and Ohmeda 3700 pulse oximeters. *J. Clin. Monit. Comput.* **4**, 31–36 (1987).
8. Larsen, V. H., Christensen, P.-H., Oxhøj, H. & Brask, T. Impedance pneumography for long-term monitoring of respiration during sleep in adult males. *Clin. Physiol.* **4**, 333–342 (1984).
